# Supplementary material for: GAK antagonises ROCK-dependent regulation of actomyosin dynamics
Source: J Cell Sci. 2026 Apr 17;139(7):jcs264117. doi: 10.1242/jcs.264117 (PMC13120681; doi:10.1242/jcs.264117)
Supplement: Supplementary information [file joces-139-264117-s1.pdf]

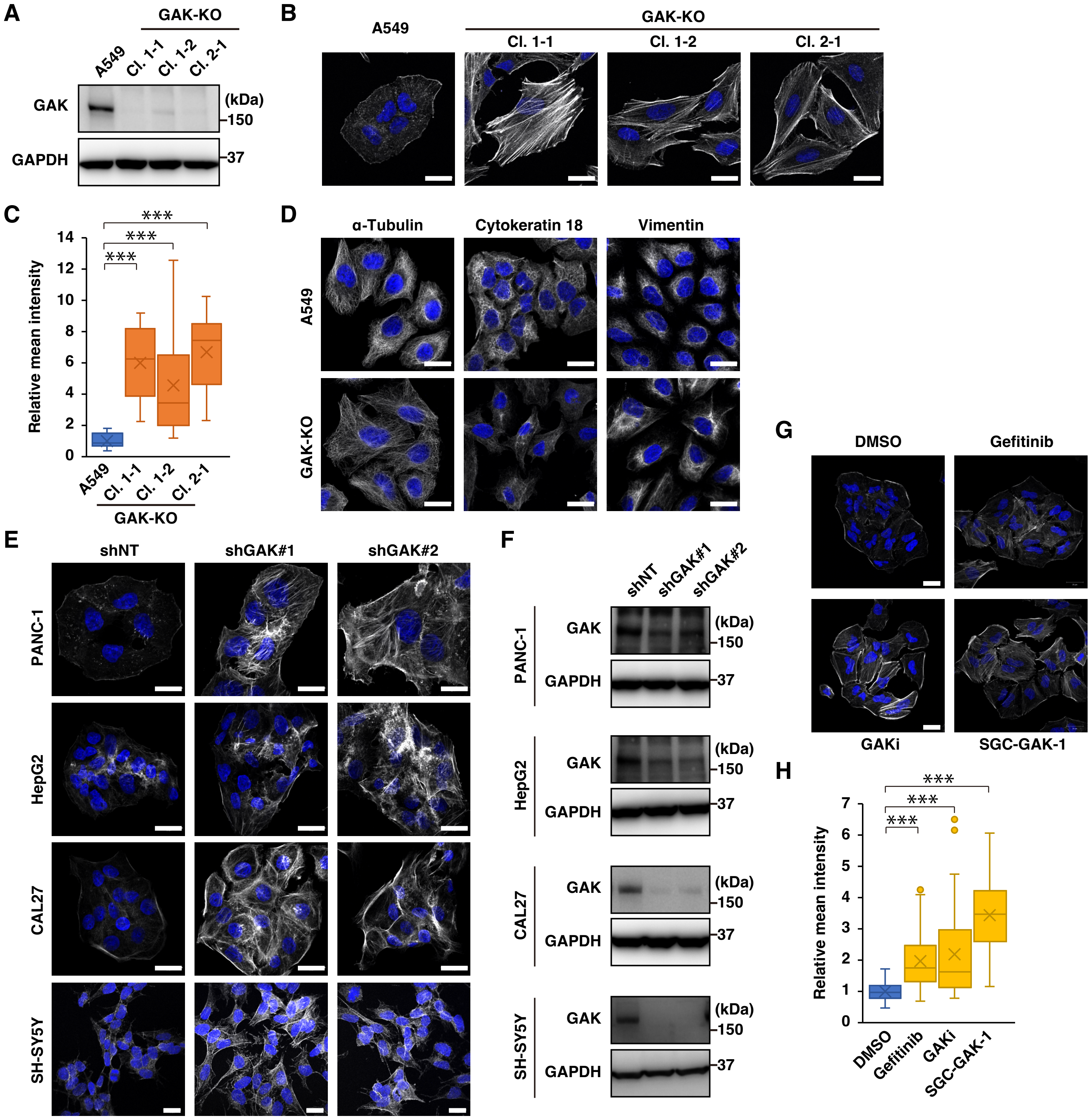

Fig. S1. GAK disruption induces stress fibre formation.

(A) IB analysis of GAK expression in A549 WT and GAK-KO cells (Clones 1-1, 1-2, and 2-1). (B) F-actin (phalloidin) staining in A549 WT and GAK-KO cells (Clones 1-1, 1-2, and 2-1). Representative images are shown. Scale bar, 20 µm. (C) Quantification of the phalloidin mean intensity. At least 30 cells per condition were quantified from three independent experiments and are presented in box plots. The box extends from the lower to the upper quartile; the middle line indicates the median; the X indicates the mean; and the whiskers represent the minimum to maximum values, except for outliers, which are shown as dots. ***$p < 0.001$ (one-way ANOVA followed by Tukey–Kramer post hoc test). (D) IF microscopy analysis of A549 WT and GAK-KO cells stained for α-tubulin, cytokeratin 18, or vimentin. Representative images are shown. Scale bar, 20 µm. (E) F-actin (phalloidin) staining in PANC-1, HepG2, CAL27, and SH-SY5Y cells transduced with non-targeting shRNA (shNT) or GAK-targeted shRNA (shGAK#1 and shGAK#2). Representative images are shown. Scale bar, 20 µm. (F) IB analysis of PANC-1, HepG2, CAL27, and SH-SY5Y cells transduced with shNT, shGAK#1, or shGAK#2. (G) F-actin (phalloidin) staining in A549 cells treated with DMSO, gefitinib (30 µM), GAK inhibitor (30 µM), or SGC-GAK-1 (10 µM) for 3 h. Representative images are shown. Scale bar, 20 µm. (H) Quantification of phalloidin mean intensity. At least 30 cells per condition were quantified from three independent experiments and are presented in box plots. The box extends from the lower to the upper quartile; the middle line indicates the median; the X indicates the mean; and the whiskers represent the minimum to maximum values, except for outliers, which are shown as dots. ***$p < 0.001$ (one-way ANOVA followed by Tukey–Kramer post hoc test).

Journal of Cell Science • Supplementary information

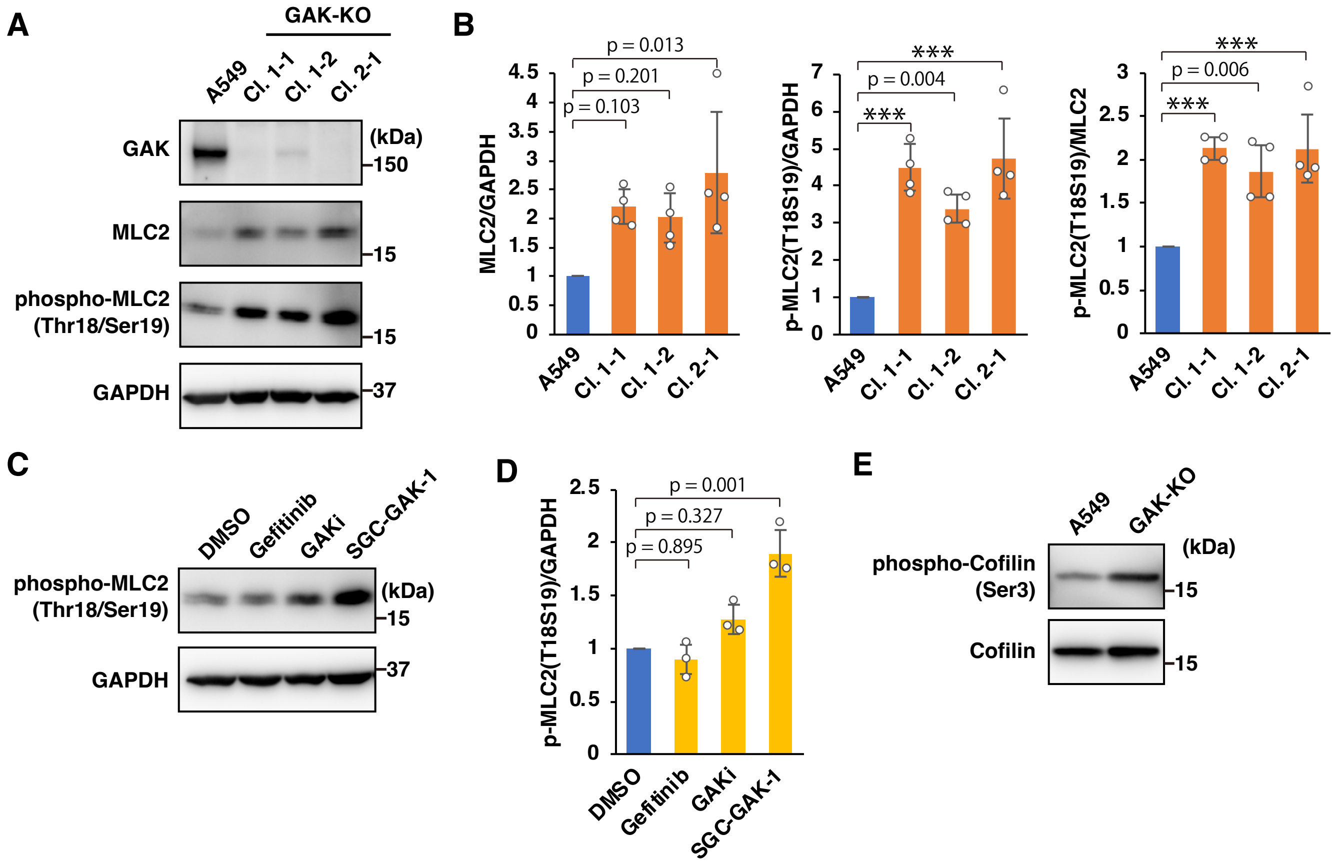

Fig. S2. GAK disruption promotes the phosphorylation of MLC and cofilin.

(A) IB analysis of MLC2 and phosphorylated MLC2 in A549 WT and GAK-KO cells (Clones 1-1, 1-2, and 2-1). Representative blots are shown. (B) Quantification of the relative band intensity of total MLC2 and phosphorylated MLC2 (Thr18/Ser19), normalised to GAPDH or total MLC2. Data are presented as mean ± SD (n = 4). ***$p < 0.001$ (one-way ANOVA followed by Tukey–Kramer post hoc test). (C) IB analysis of phosphorylated MLC2 (Thr18/Ser19) in A549 cells treated with DMSO, gefitinib (30 μM), GAK inhibitor (30 μM), or SGC-GAK-1 (10 μM) for 1 h. Representative blots are shown. (D) Quantification of the relative band intensity of phosphorylated MLC2 (Thr18/Ser19), normalised to GAPDH. Data are presented as mean ± SD (n = 3; one-way ANOVA followed by Tukey–Kramer post hoc test). (E) IB analysis of cofilin and phosphorylated cofilin (Ser3) in A549 WT and GAK-KO cells. Representative blots are shown.

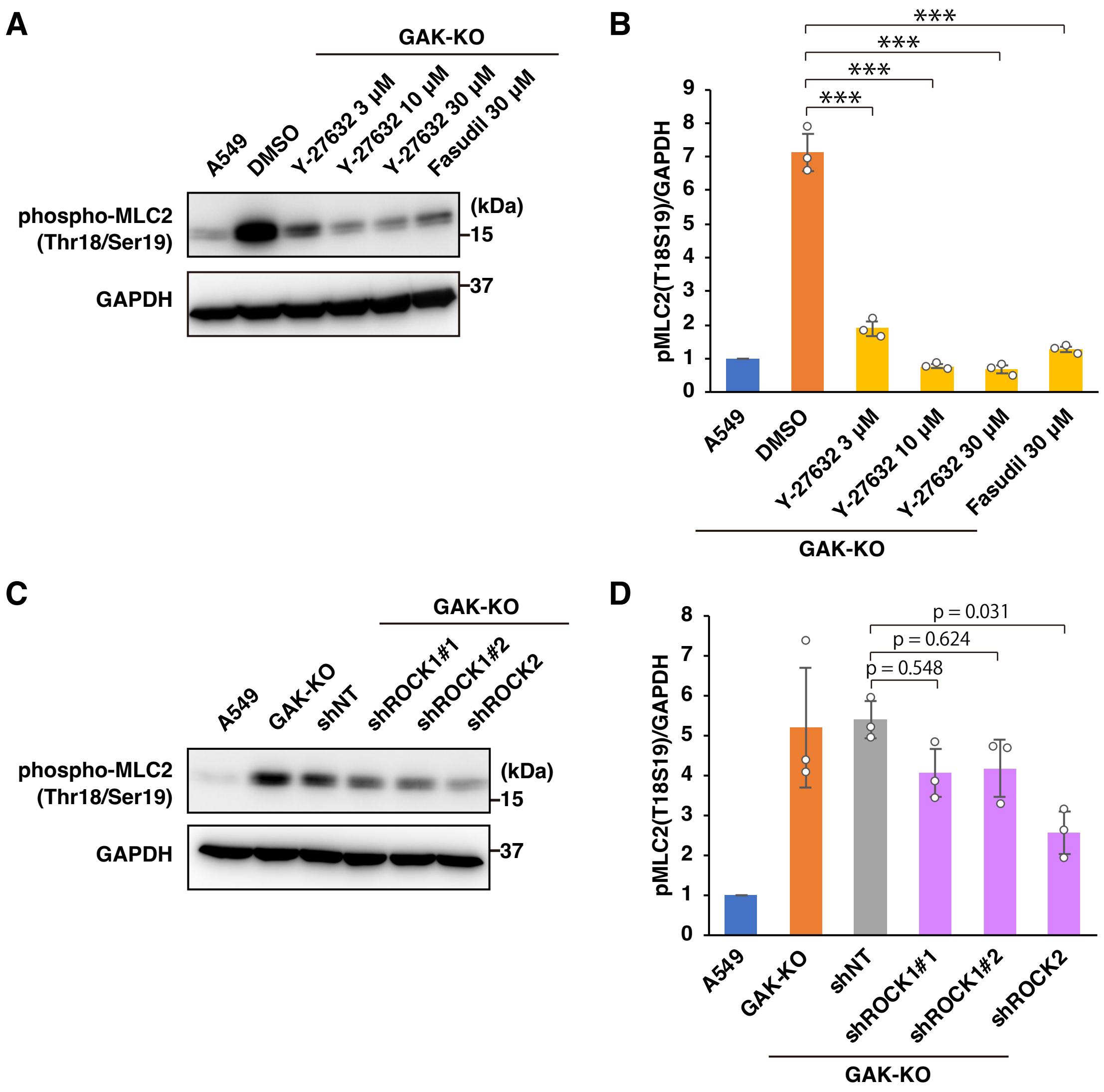

Fig. S3. ROCK inhibition attenuates MLC phosphorylation in GAK-KO cells.

(A) IB analysis of phosphorylated MLC2 (Thr18/Ser19) in A549 WT and GAK-KO cells. GAK-KO cells were treated with DMSO, Y-27632 (3–30 μM), or fasudil (30 μM) for 24 h. Representative blots are shown. (B) Quantification of the relative band intensity of phosphorylated MLC2 (Thr18/Ser19), normalised to GAPDH. Data are presented as mean ± SD (n = 3). ***$p < 0.001$ (one-way ANOVA followed by Tukey–Kramer post hoc test). (C) IB analysis of phosphorylated MLC2 (Thr18/Ser19) in A549 WT and GAK-KO cells. GAK-KO cells were transduced with non-targeting shRNA (GAK-KO/shNT), ROCK1-targeted shRNA (GAK-KO/shROCK1#1 and GAK-KO/shROCK1#2), or ROCK2-targeted shRNA (GAK-KO/shROCK2). Representative blots are shown. (D) Quantification of the relative band intensity of phosphorylated MLC2 (Thr18/Ser19), normalised to GAPDH. Data are presented as mean ± SD (n = 3; one-way ANOVA followed by Tukey–Kramer post hoc test).

Journal of Cell Science • Supplementary information

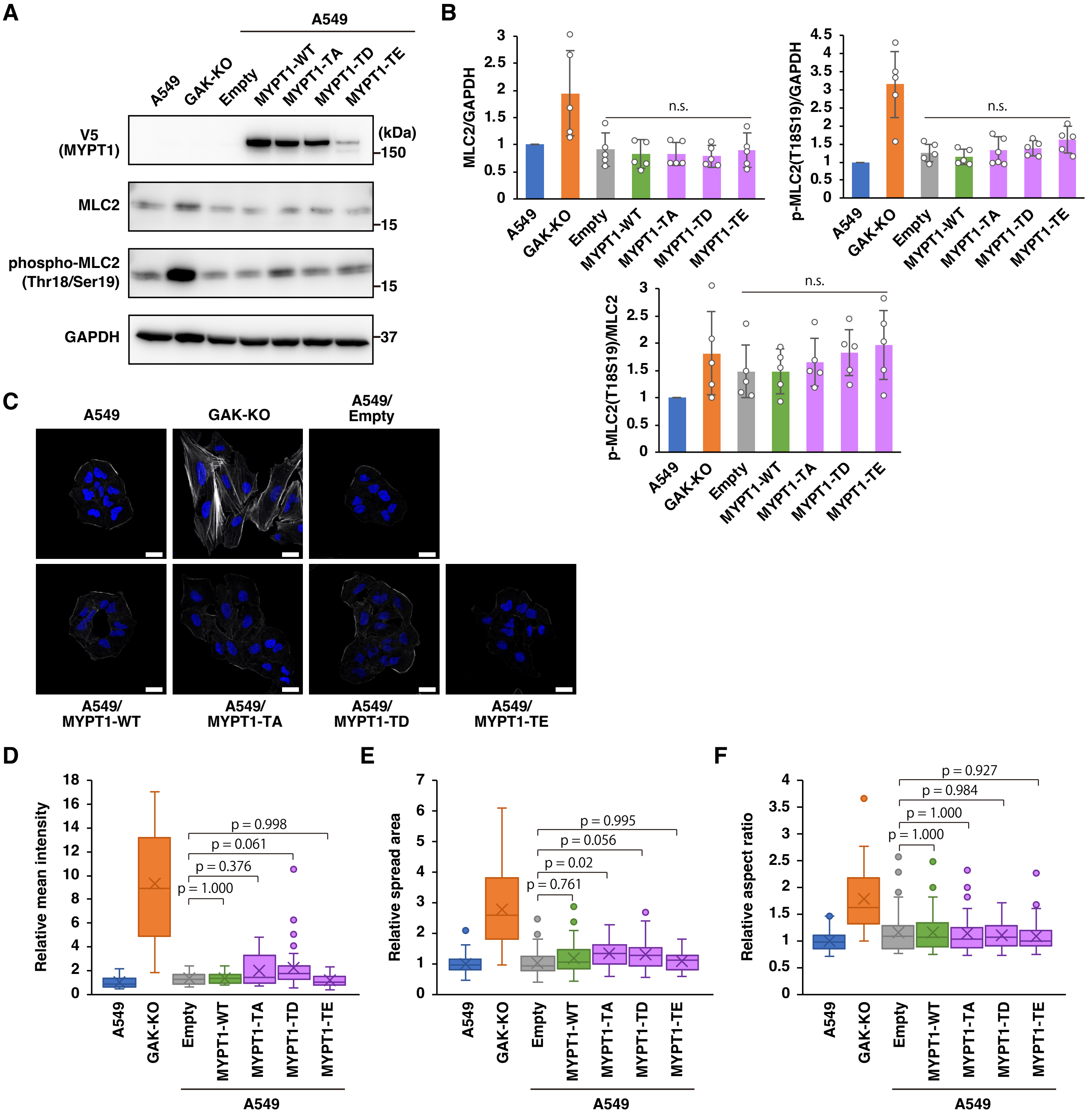

Fig. S4. Forced expression of MYPT1 mutants has little effect on A549 cells.

(A) IB analysis of total MLC2 and phosphorylated MLC2 (Thr18/Ser19) in A549 WT and GAK-KO cells. A549 WT cells were transduced with an empty vector (Empty), MYPT1-WT, or MYPT1 mutants (MYPT1-T443A, MYPT1-T443D, or MYPT1-T443E). Representative blots are shown. (B) Quantification of the relative band intensity of total MLC2 and phosphorylated MLC2 (Thr18/Ser19), normalised to GAPDH or total MLC2. Data are presented as mean ± SD (n = 5; one-way ANOVA followed by Tukey–Kramer post hoc test). (C) F-actin (phalloidin) staining in A549 WT and GAK-KO cells. A549 WT cells were transduced with an empty vector (Empty), MYPT1-WT, or MYPT1 mutants (MYPT1-T443A, MYPT1-T443D, or MYPT1-T443E). Representative images are shown. Scale bar, 20 μm. (D-F) Quantification of phalloidin mean intensity (D), cell spread area (E), and cell aspect ratio (F). At least 30 cells per condition were quantified from three independent experiments and are presented in box plots. The box extends from the lower to the upper quartile; the middle line indicates the median; the X indicates the mean; and the whiskers represent the minimum to maximum values, except for outliers, which are shown as dots. ***$p < 0.001$ (one-way ANOVA followed by Tukey–Kramer post hoc test).

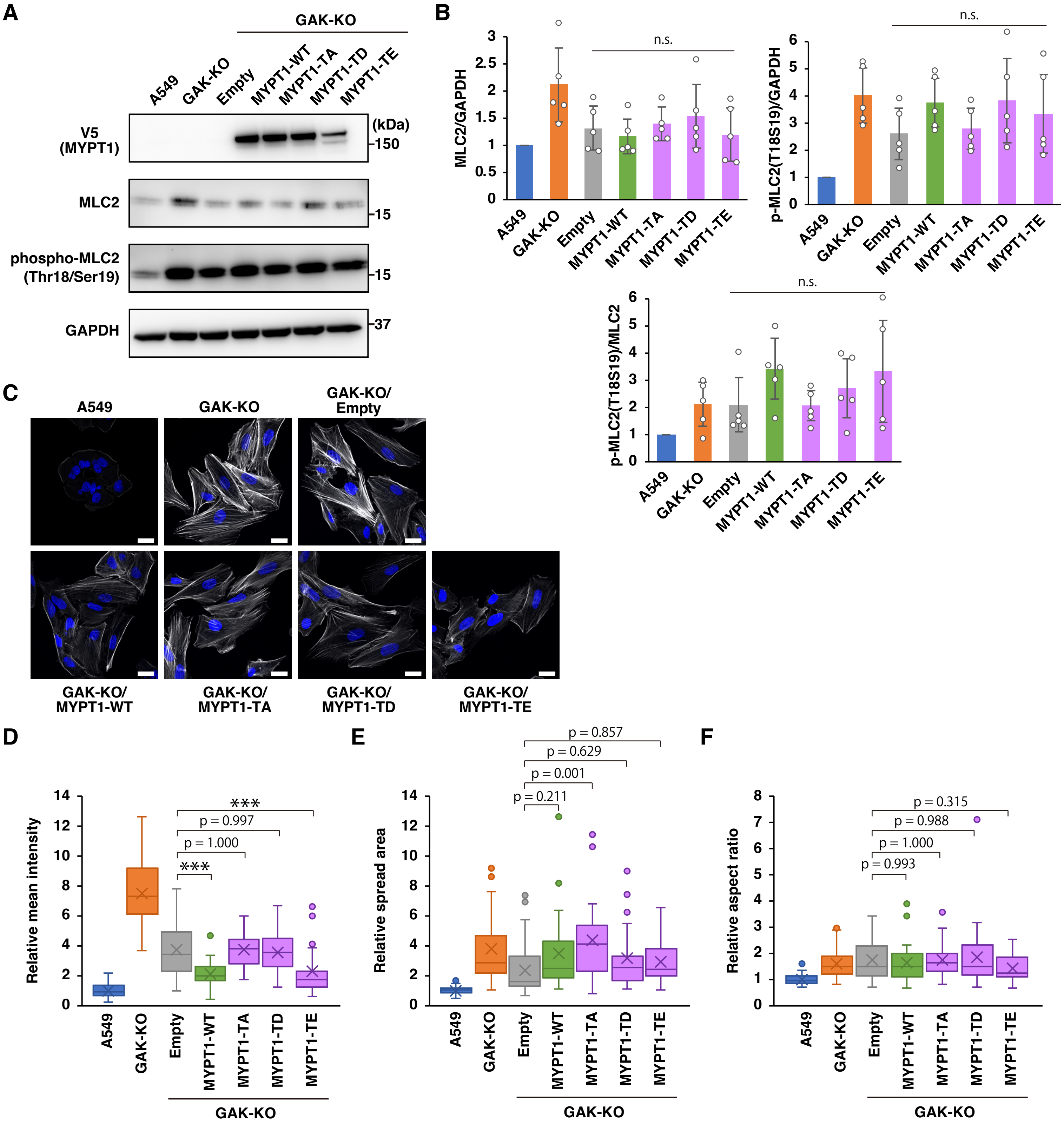

Fig. S5. Forced expression of a MYPT1 phosphorylation-mimicking mutant attenuates stress fiber formation in GAK-KO cells.

(A) IB analysis of total MLC2 and phosphorylated MLC2 (Thr18/Ser19) in A549 WT and GAK-KO cells. GAK-KO cells were transduced with an empty vector (Empty), MYPT1-WT, or MYPT1 mutants (MYPT1-T443A, MYPT1-T443D, or MYPT1-T443E). Representative blots are shown. (B) Quantification of the relative band intensity of total MLC2 and phosphorylated MLC2 (Thr18/Ser19), normalised to GAPDH or total MLC2. Data are presented as mean ± SD (n = 5; one-way ANOVA followed by Tukey–Kramer post hoc test). (C) F-actin (phalloidin) staining in A549 WT and GAK-KO cells. GAK-KO cells were transduced with an empty vector (Empty), MYPT1-WT, or MYPT1 mutants (MYPT1-T443A, MYPT1-T443D, or MYPT1-T443E). Representative images are shown. Scale bar, 20 μm. (D-F) Quantification of phalloidin mean intensity (D), cell spread area (E), and cell aspect ratio (F). At least 30 cells per condition were quantified from three independent experiments and are presented in box plots. The box extends from the lower to the upper quartile; the middle line indicates the median; the X indicates the mean; and the whiskers represent the minimum to maximum values, except for outliers, which are shown as dots. ***$p < 0.001$ (one-wayANOVA followed by Tukey–Kramer post hoc test).

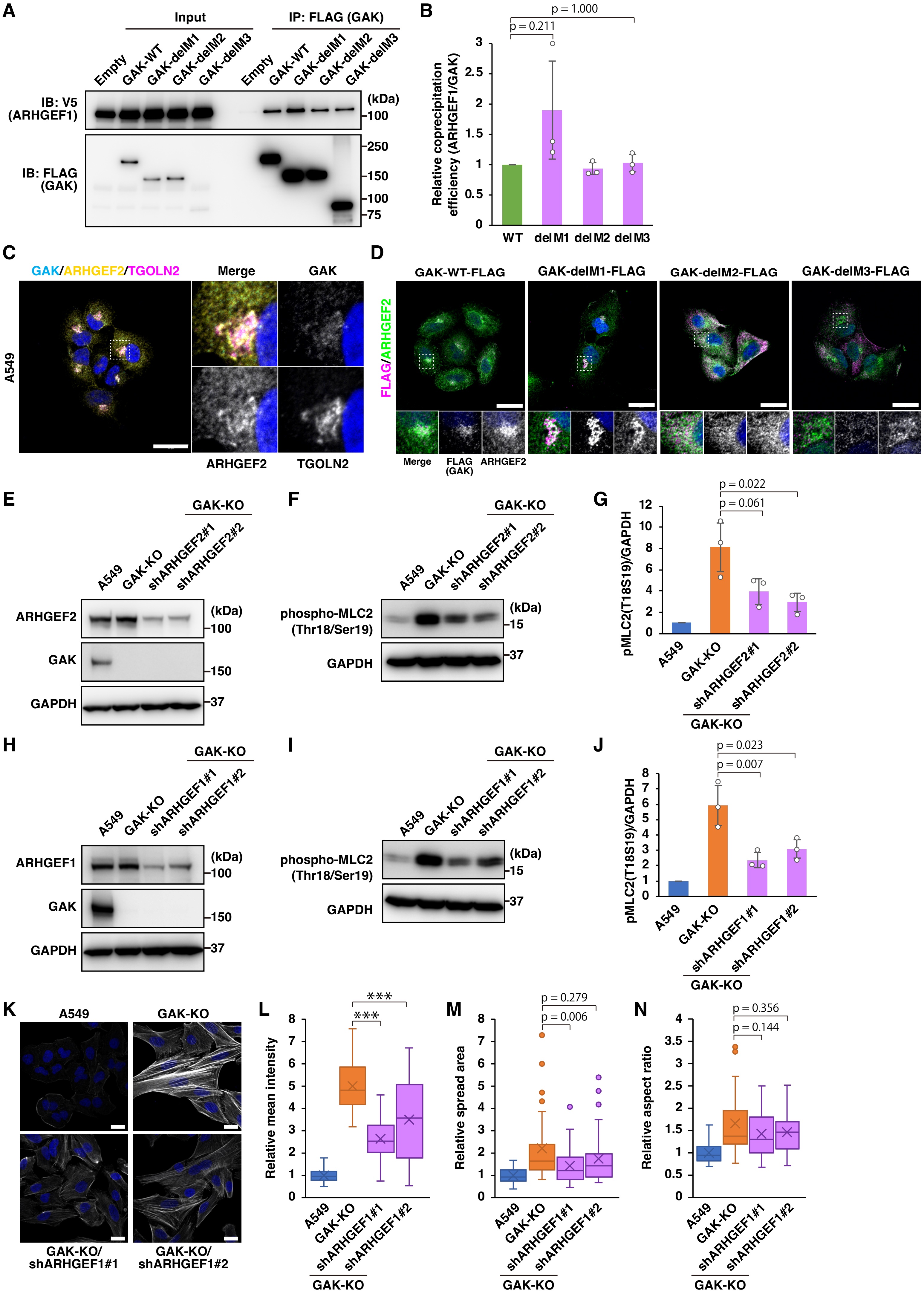

Journal of Cell Science • Supplementary information

Fig. S6. The IDR of GAK is involved in actomyosin regulation.

(A) Immunoprecipitation with anti-FLAG antibody was performed on lysates from 293T cells overexpressing FLAG-tagged GAK (WT or mutants) and V5-tagged ARHGEF1, followed by IB analysis with anti-V5 and anti-FLAG antibodies. Representative blots are shown. (B) Quantification of the relative co-precipitation efficiency of ARHGEF1 with GAK (WT or mutants). Data are presented as mean ± SD (n = 3 ; one-way ANOVA followed by Tukey–Kramer post hoc test). (C) IF images of A549 cells stained for GAK (cyan in merge), ARHGEF2 (yellow in merge), TGOLN2 (magenta in merge), and DNA (DAPI; blue in merge). The dashed boxed regions are shown at a high magnification (x4) on the right. Representative images are shown. Scale bar, 20 μm. (D) IF images of GAK-KO cells transduced with FLAG-tagged GAK-WT, or GAK mutants (GAK-delM1, GAK-delM2, or GAK-delM3). These cells stained for GAK-FLAG (magenta in merge), ARHGEF2 (green in merge), and DNA (DAPI; blue in merge). The dashed boxed regions are shown at a high magnification (x3) at the bottom. Representative images are shown. Scale bar, 20 μm. (E) IB analysis of ARHGEF2 expression in A549 WT, GAK-KO, GAK-KO/shARHGEF2#1, and GAK-KO/shARHGEF2#2 cells. (F) IB analysis of phosphorylated MLC2 (Thr18/Ser19) in A549 WT and GAK-KO cells. GAK-KO cells were transduced with ARHGEF2-targeted shRNA (GAK-KO/sh ARHGEF2#1 and GAK-KO/shARHGEF2#2). Representative blots are shown. (G) Quantification of the relative band intensity of phosphorylated MLC2 (Thr18/Ser19), normalised to GAPDH for IB data in (F). Data are presented as mean ± SD (n = 3; one-way ANOVA followed by Tukey–Kramer post hoc test). (H) IB analysis of ARHGEF1 expression in A549 WT, GAK-KO, GAK-KO/shARHGEF1#1, and GAK-KO/shARHGEF1#2 cells. (I) IB analysis of phosphorylated MLC2 (Thr18/Ser19) in A549 WT and GAK-KO cells. GAK-KO cells were transduced with ARHGEF1-targeted shRNA (GAK-KO/sh ARHGEF1#1 and GAK-KO/shARHGEF1#2). Representative blots are shown. (J) Quantification of the relative band intensity of phosphorylated MLC2 (Thr18/Ser19), normalised to GAPDH for IB data in (I). Data are presented as mean ± SD (n = 3; one-way ANOVA followed by Tukey–Kramer post hoc test). (K) F-actin (phalloidin) staining in A549 WT, GAK-KO, and GAK-KO cells transduced with ARHGEF1-targeted shRNA (GAK-KO/shARHGEF1#1 and GAK-KO/shARHGEF1#2). Representative images are shown. Scale bar, 20 μm. (L-N) Quantification of phalloidin mean intensity (L), cell spread area (M), and cell aspect ratio (N). At least 30 cells per condition were quantified from three independent experiments and are presented in box plots. The box extends from the lower to the upper quartile; the middle line indicates the median; the X indicates the mean; and the whiskers represent the minimum to maximum values, except for outliers, which are shown as dots. ***p < 0.001 (one-way ANOVA followed by Tukey–Kramer post hoc test).

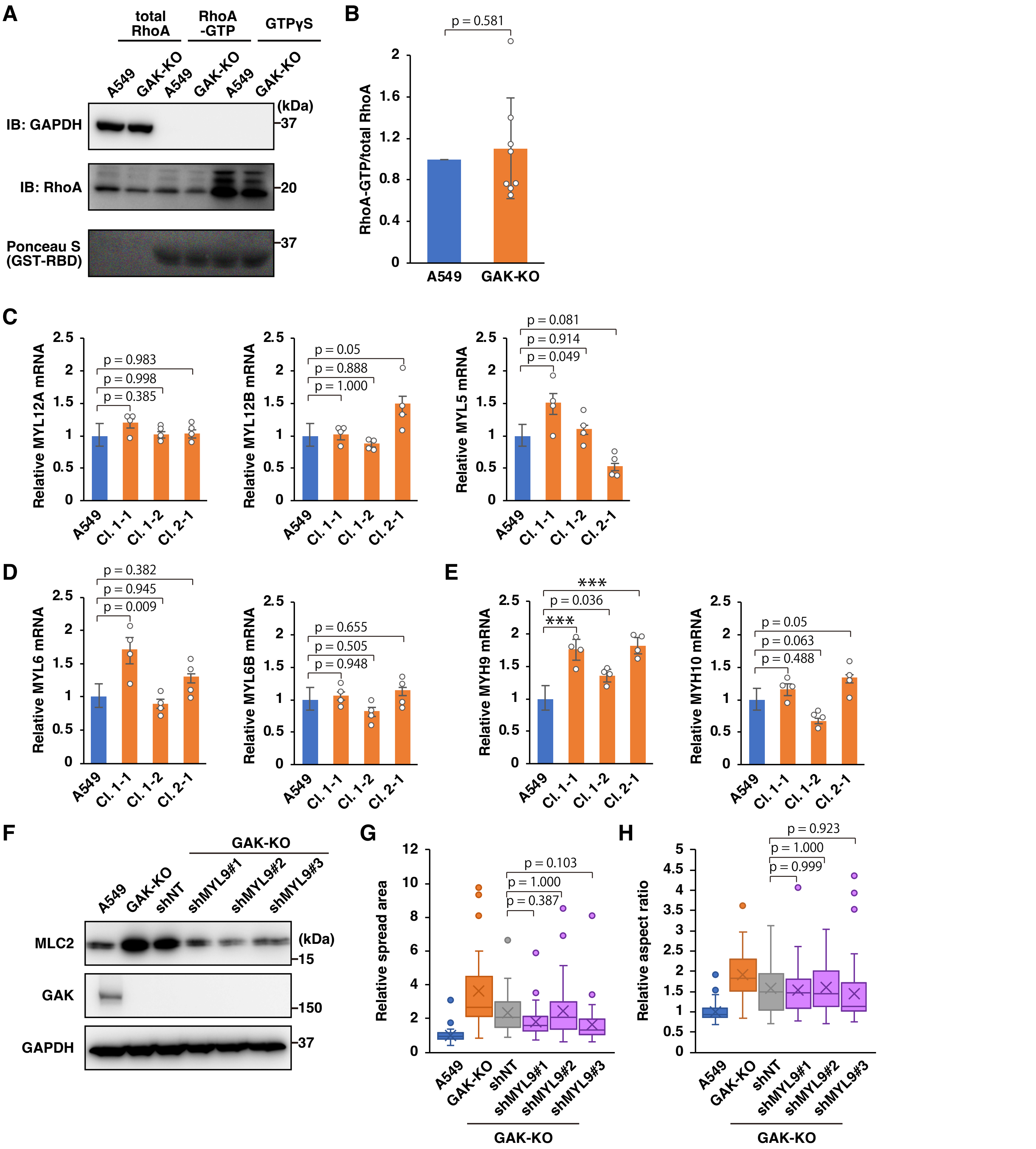

Fig. S7. The IDR of GAK is involved in MLC gene expression.

(A) GST-RBD pull-down assay was performed on lysates from A549 WT or GAK-KO treated with or without GTPγS (0.1 mM, 15 min), followed by IB analysis with anti-RhoA and anti-GAPDH antibodies. Ponceau S staining was used as a loading control of GST-RBD. Representative blots are shown. (B) Quantification of the relative pull-down efficiency of RhoA-GTP normalised to total RhoA. Data are presented as mean ± SD (n = 8; unpaired two-tailed Student's t-test). (C-E) RT-qPCR analysis of relative mRNA expression levels of myosin regulatory light chain genes (MYL12A, MYL12B, and MYL5) (C), myosin essential light chain genes (MYL6 and MYL6B) (D), and myosin heavy chain genes (MYH9 and MYH10) (E) in A549 WT and GAK-KO cells (Clones 1-1, 1-2, and 2-1). GAPDH was used as an internal control. Data are presented as mean ± SD (n = 4). ***$p < 0.001$ (one-way ANOVA followed by Tukey–Kramer post hoc test). (F) IB analysis of MYL9/MLC2 expression in A549 WT, GAK-KO, GAK-KO/shNT, GAK-KO/shMYL9#1, GAK-KO/shMYL9#2, and GAK-KO/shMYL9#3 cells. (G,H) Quantification of cell spread area (G) and cell aspect ratio (H) for IF data in Figure 8C. Quantification of phalloidin mean intensity. At least 30 cells per condition were quantified from three independent experiments and are presented in box plots. The box extends from the lower to the upper quartile; the middle line indicates the median; the X indicates the mean; and the whiskers represent the minimum to maximum values, except for outliers, which are shown as dots. (one-way ANOVA followed by Tukey–Kramer post hoc test).

Journal of Cell Science • Supplementary information

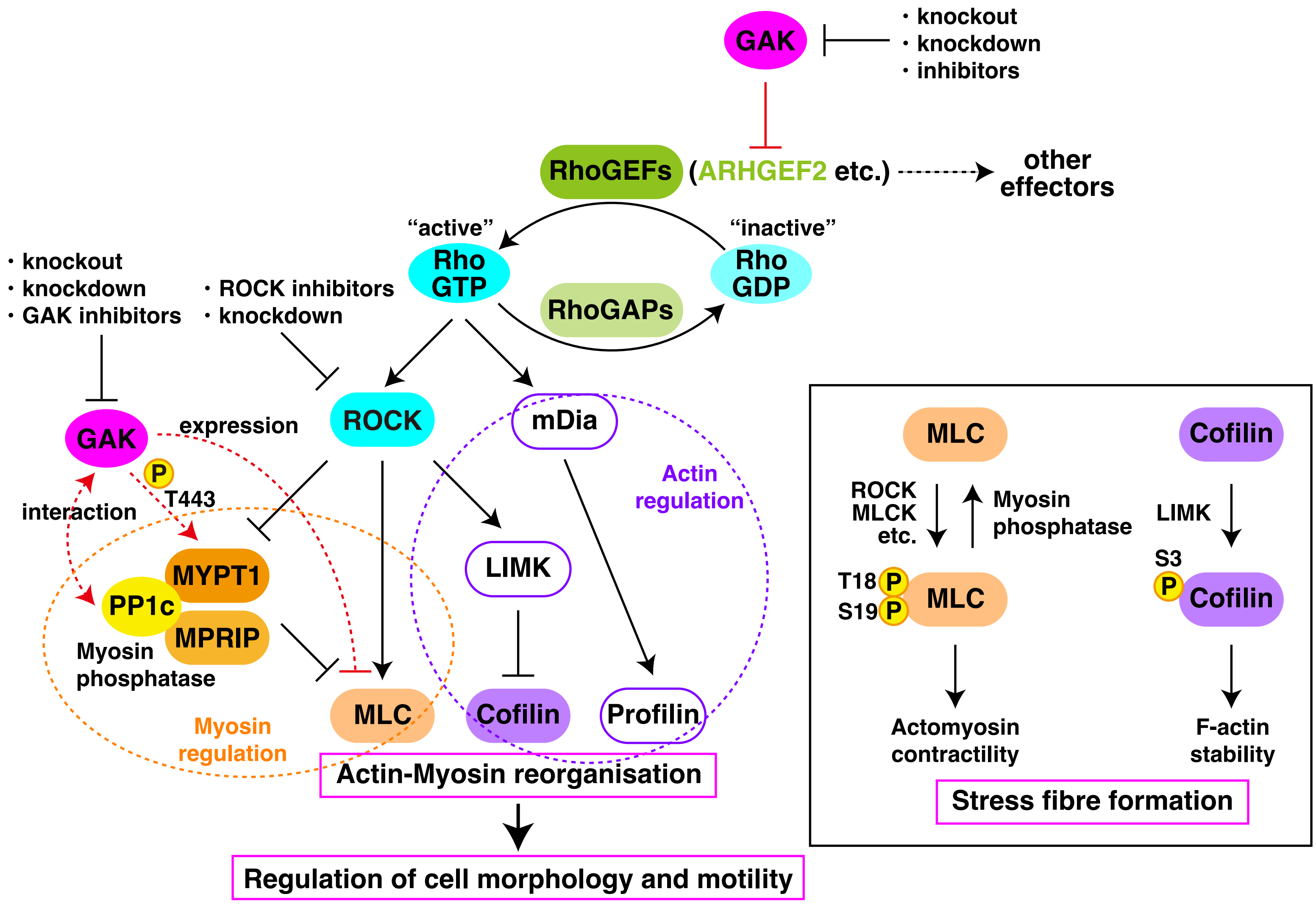

Fig. S8. Schematic diagram of ROCK-dependent regulation of actomyosin dynamics and the role of GAK in this study. RhoGEFs, including ARHGEF2, activate small G proteins, such as Rho. The GTP-bound, active form of Rho binds to ROCK or mDia to stimulate downstream effectors. ROCK-mediated MLC phosphorylation promotes actomyosin contraction. ROCK also phosphorylates the myosin phosphatase regulatory subunit MYPT1, inhibiting myosin phosphatase activity and thereby enhancing MLC phosphorylation. Additionally, ROCK activates LIMK, which phosphorylates and inactivates the actin-depolymerising protein cofilin, leading to actin filament stabilisation. In this study, we demonstrated that GAK acts antagonistically to ROCK-dependent regulation of actomyosin dynamics by suppressing ARHGEF2 activity and MLC expression. We also demonstrated that GAK phosphorylates MYPT1 at a site distinct from that targeted by ROCK and interacts with the catalytic subunit of myosin phosphatase, although the functional significance of these interactions remains unclear. LIMK, LIM domain kinase; mDia, mammalian diaphanous; MLCK, MLC kinase; RhoGAPs, Rho GTPase-activating proteins.

Fig. 1D

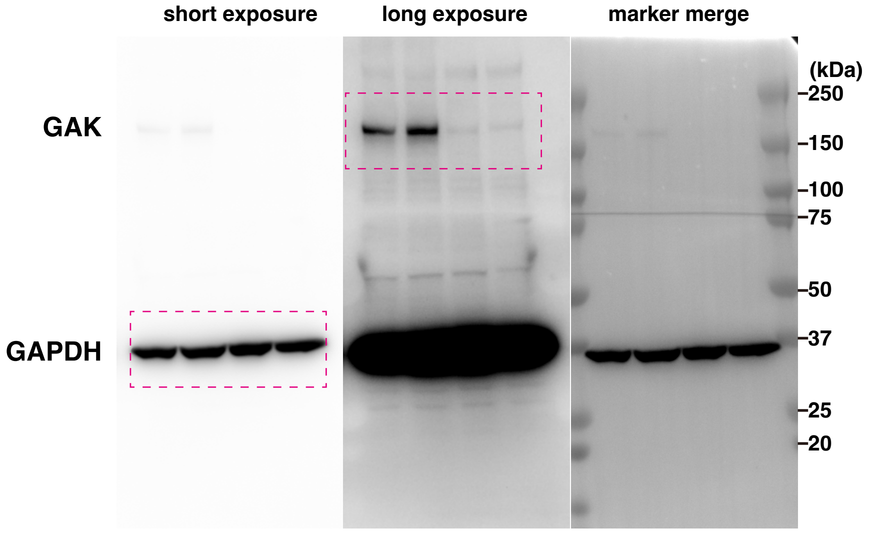

Fig. 3A

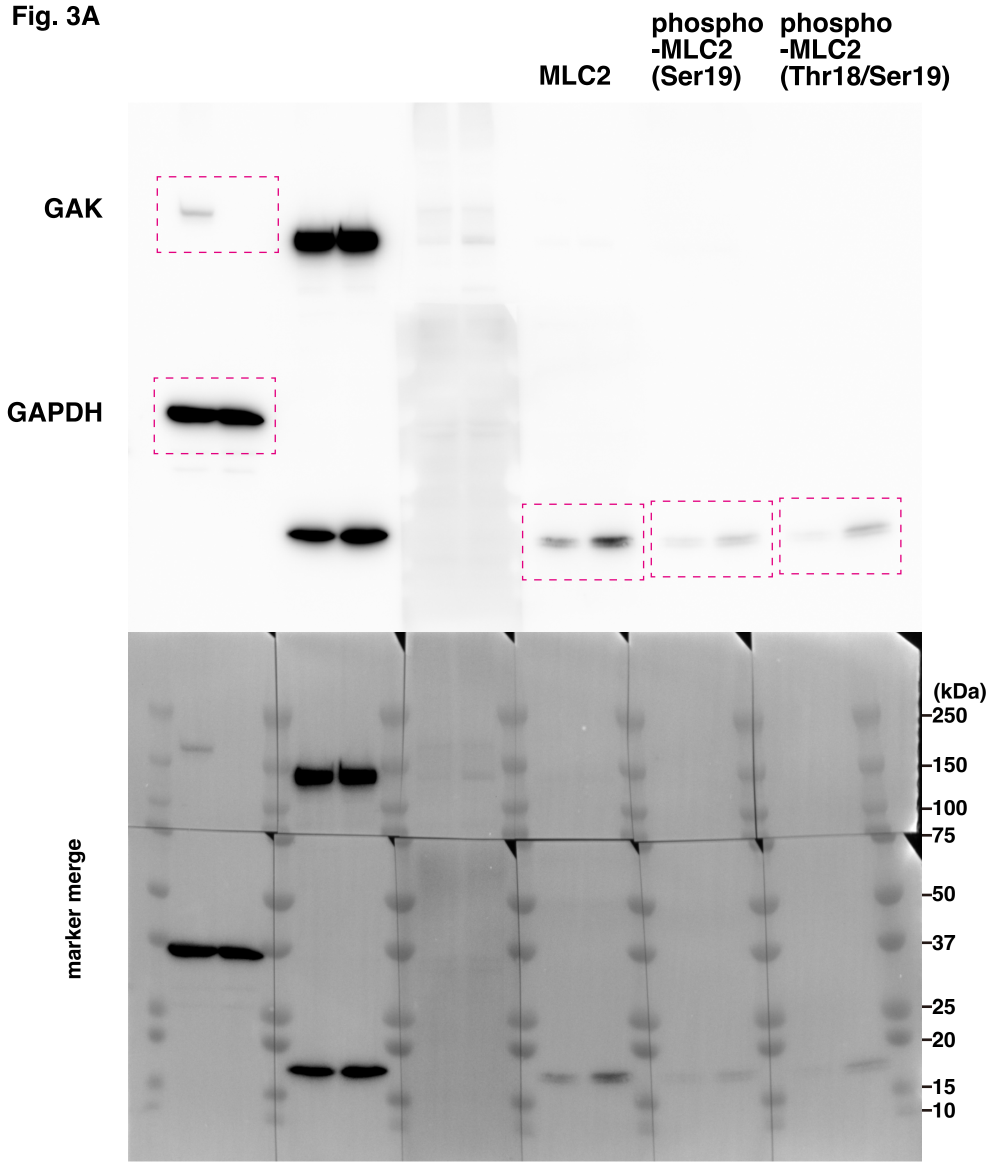

Journal of Cell Science • Supplementary information

(continued)

Fig. 3C

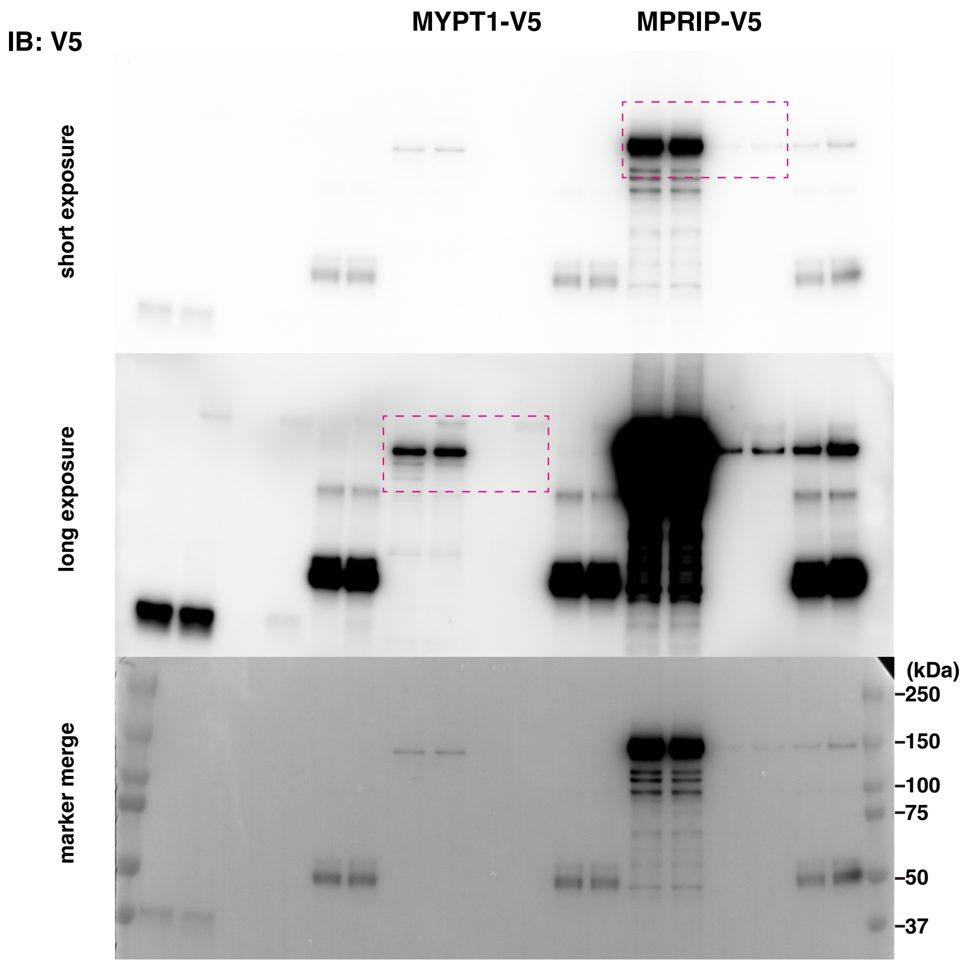

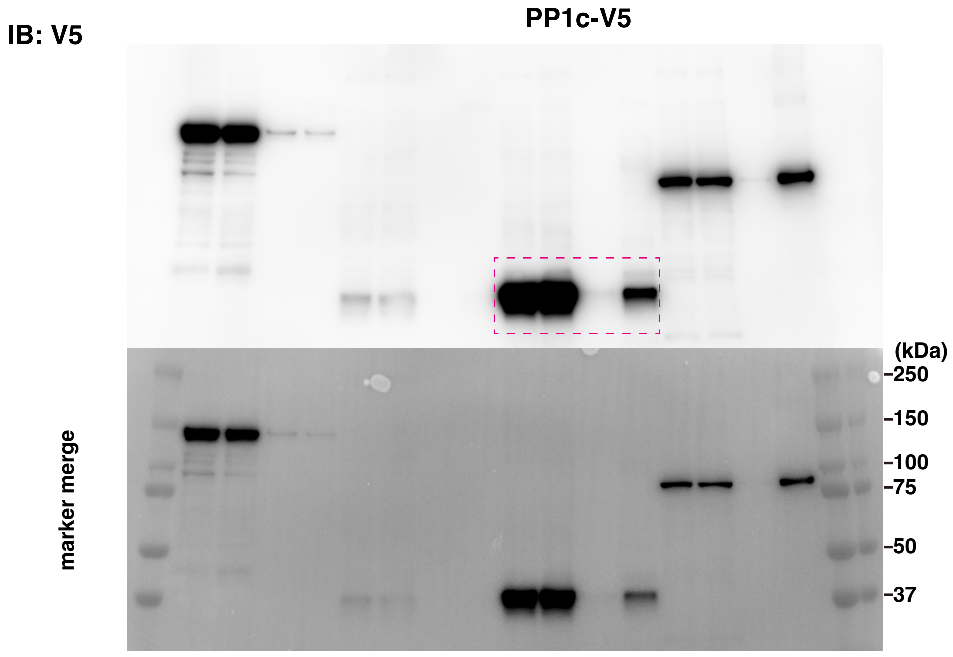

Journal of Cell Science • Supplementary information

(continued)

Fig. 3C

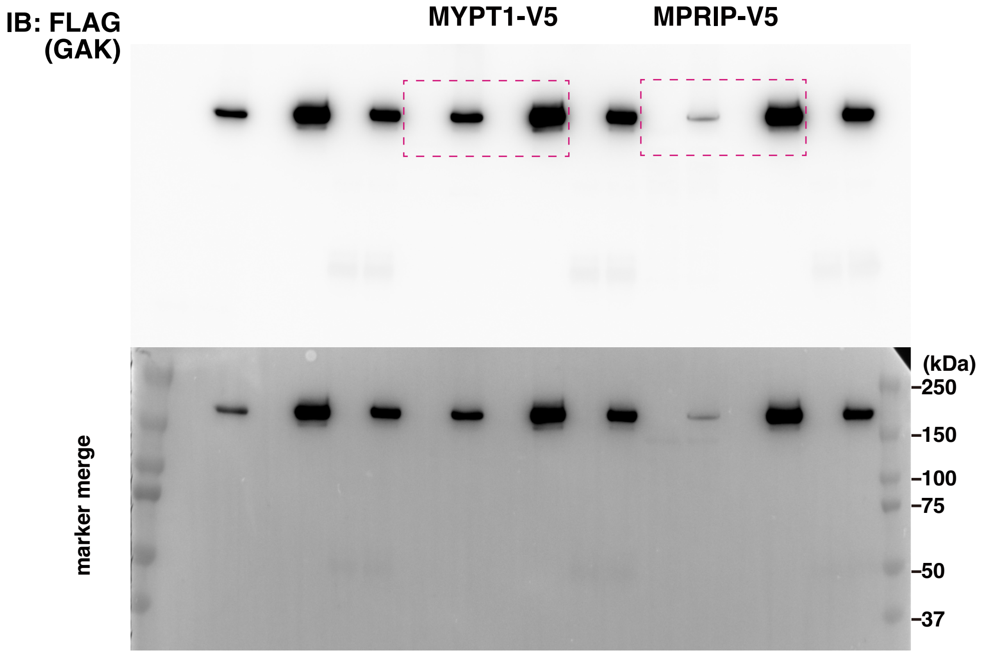

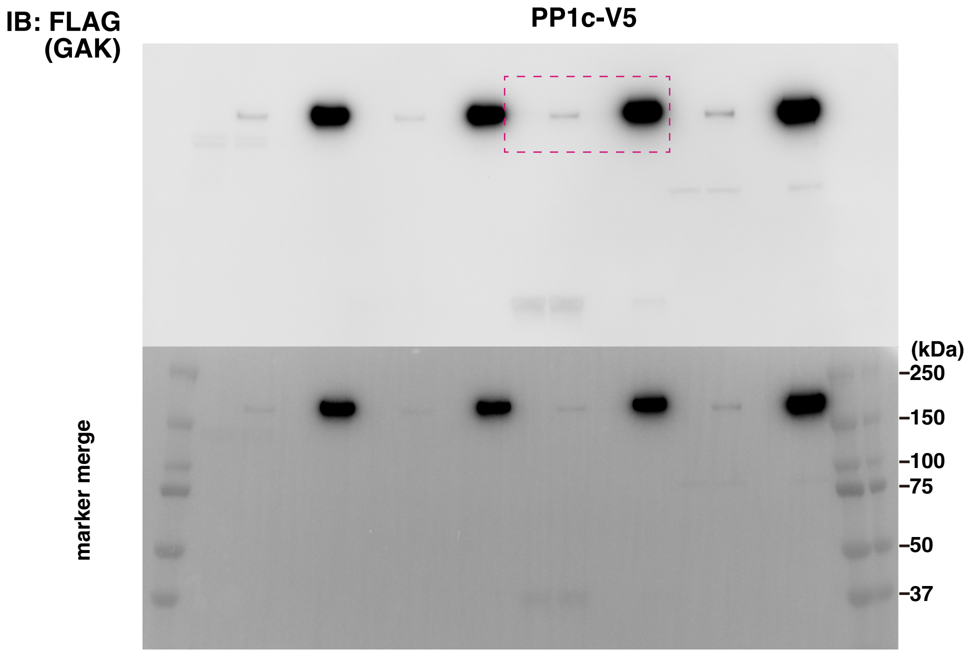

Journal of Cell Science • Supplementary information

(continued)

Fig. 4C

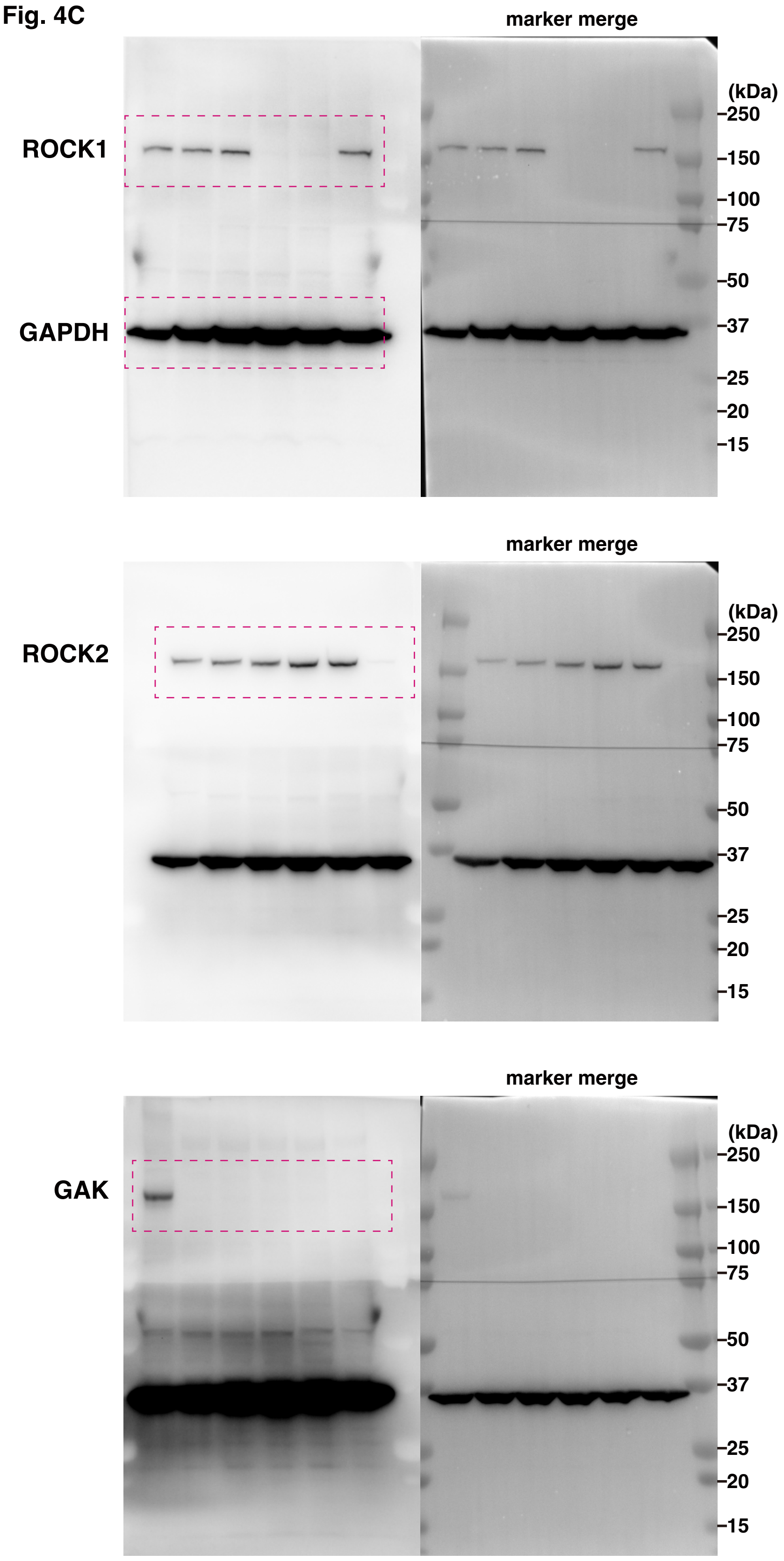

(continued)

Fig. 4D

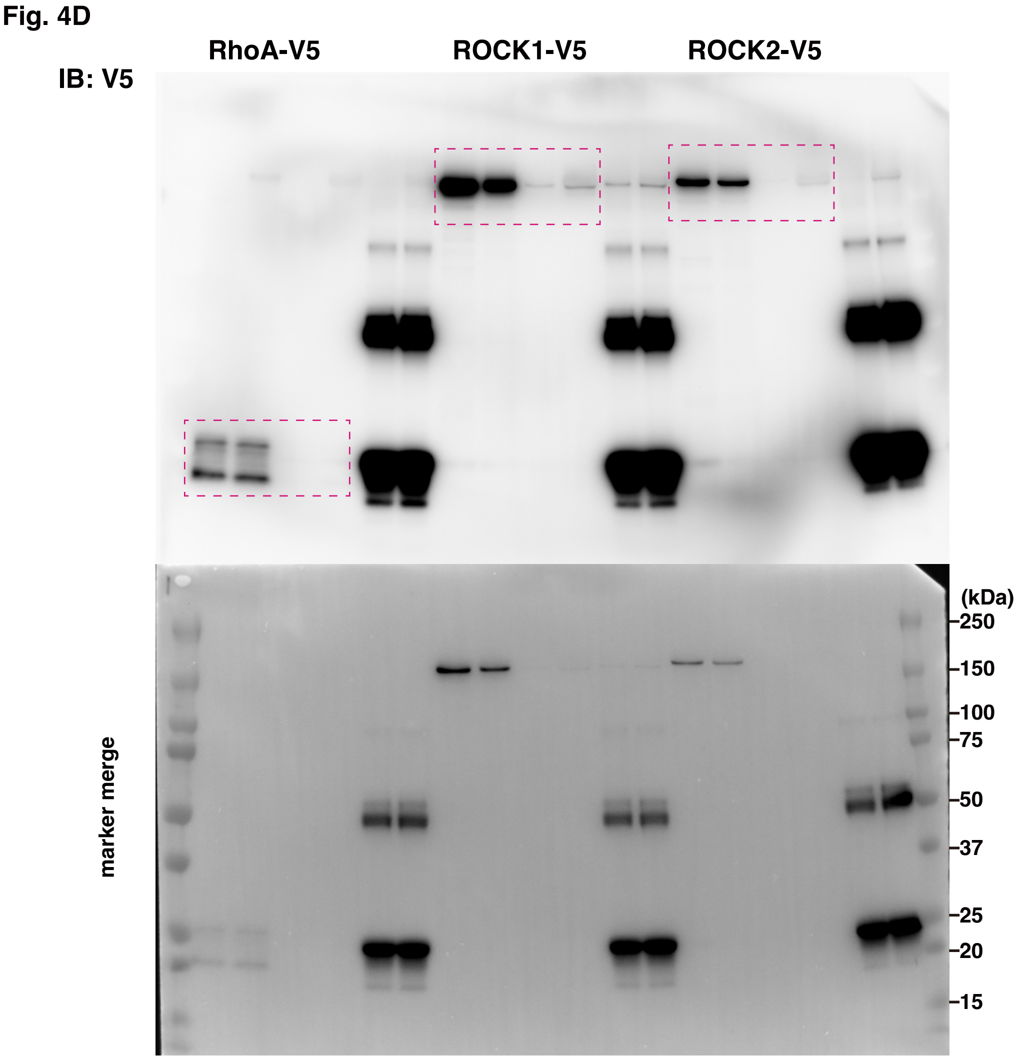

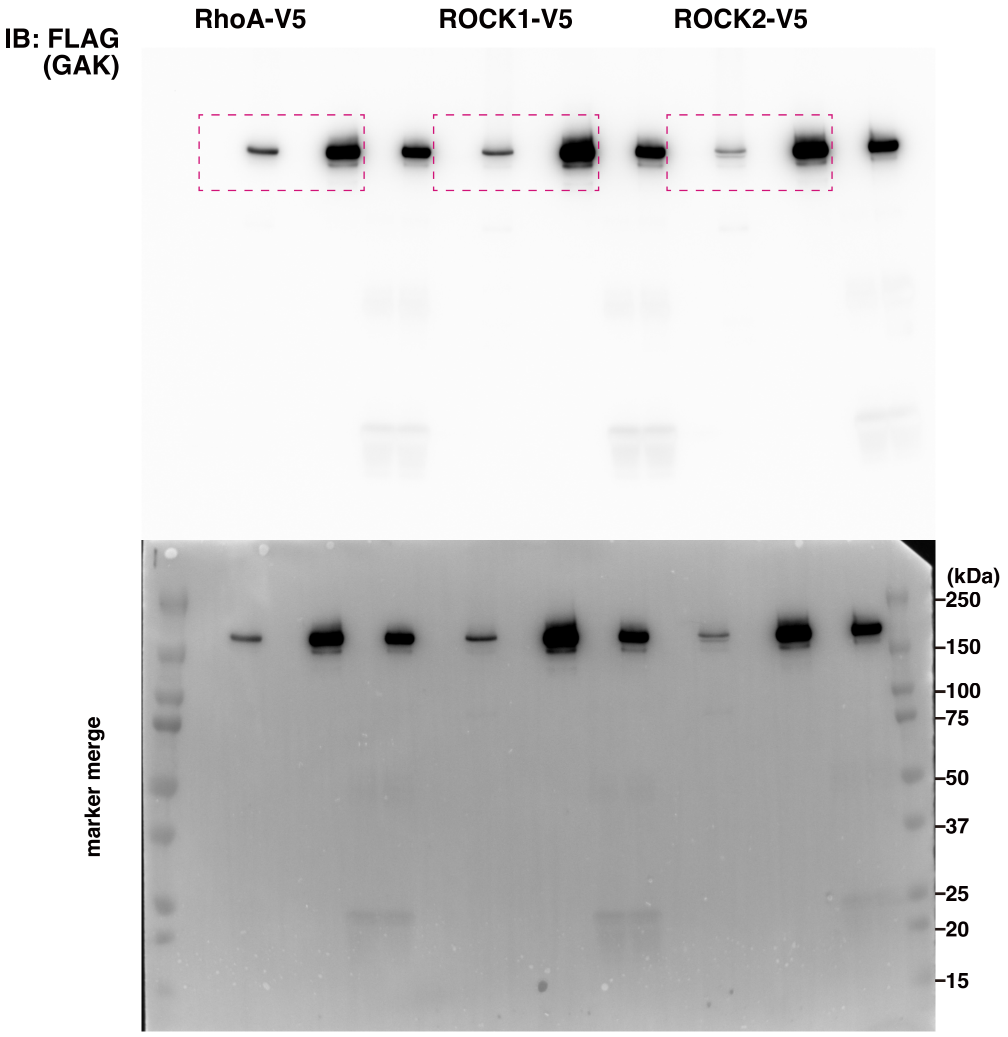

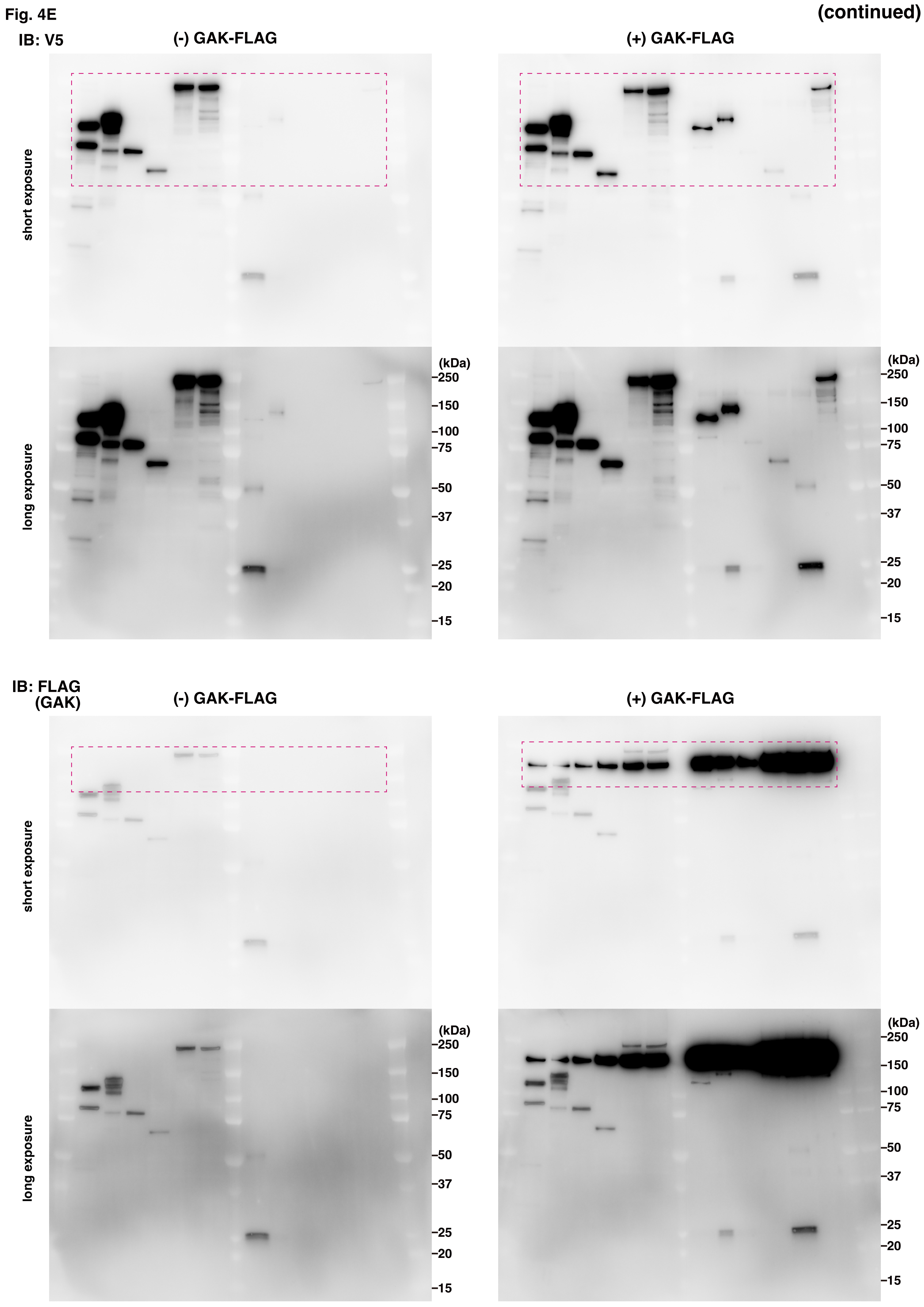

Journal of Cell Science • Supplementary information

(continued)

Fig. 7A

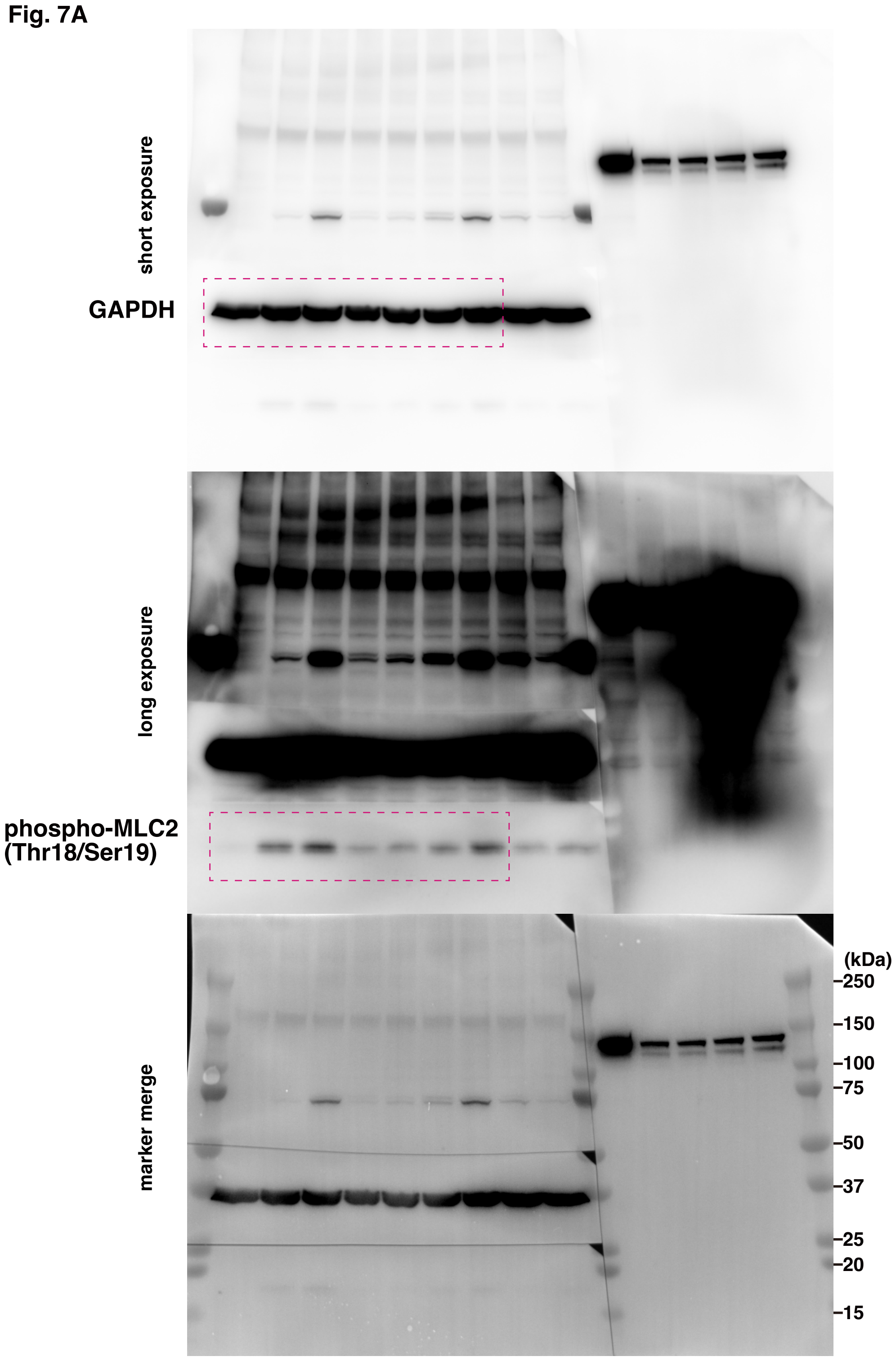

Fig. 7C

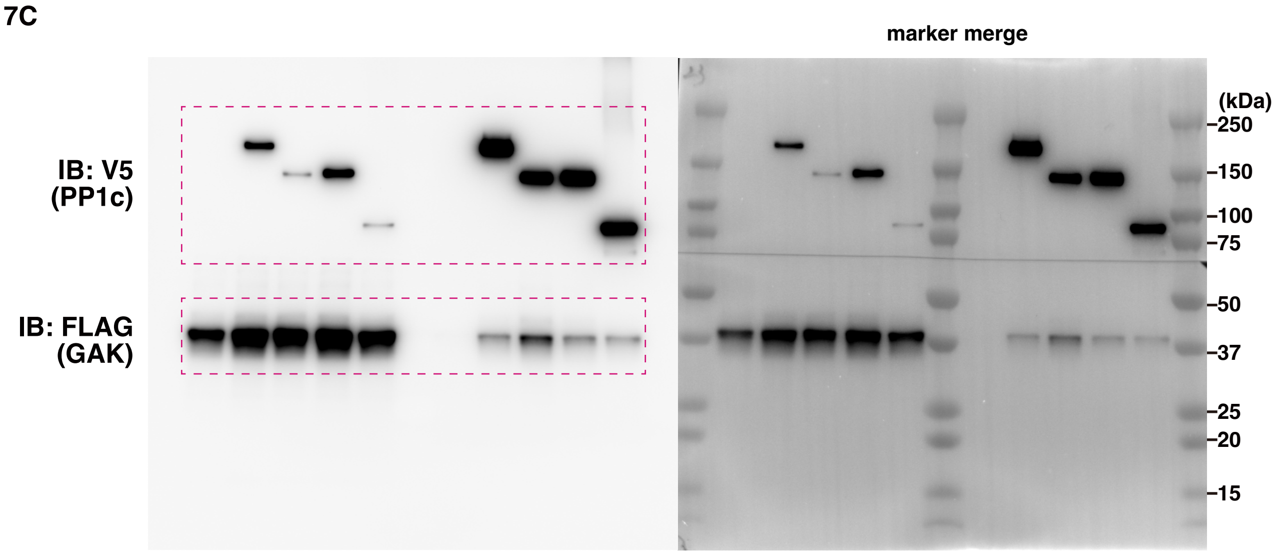

Journal of Cell Science • Supplementary information

(continued)

Fig. 7E

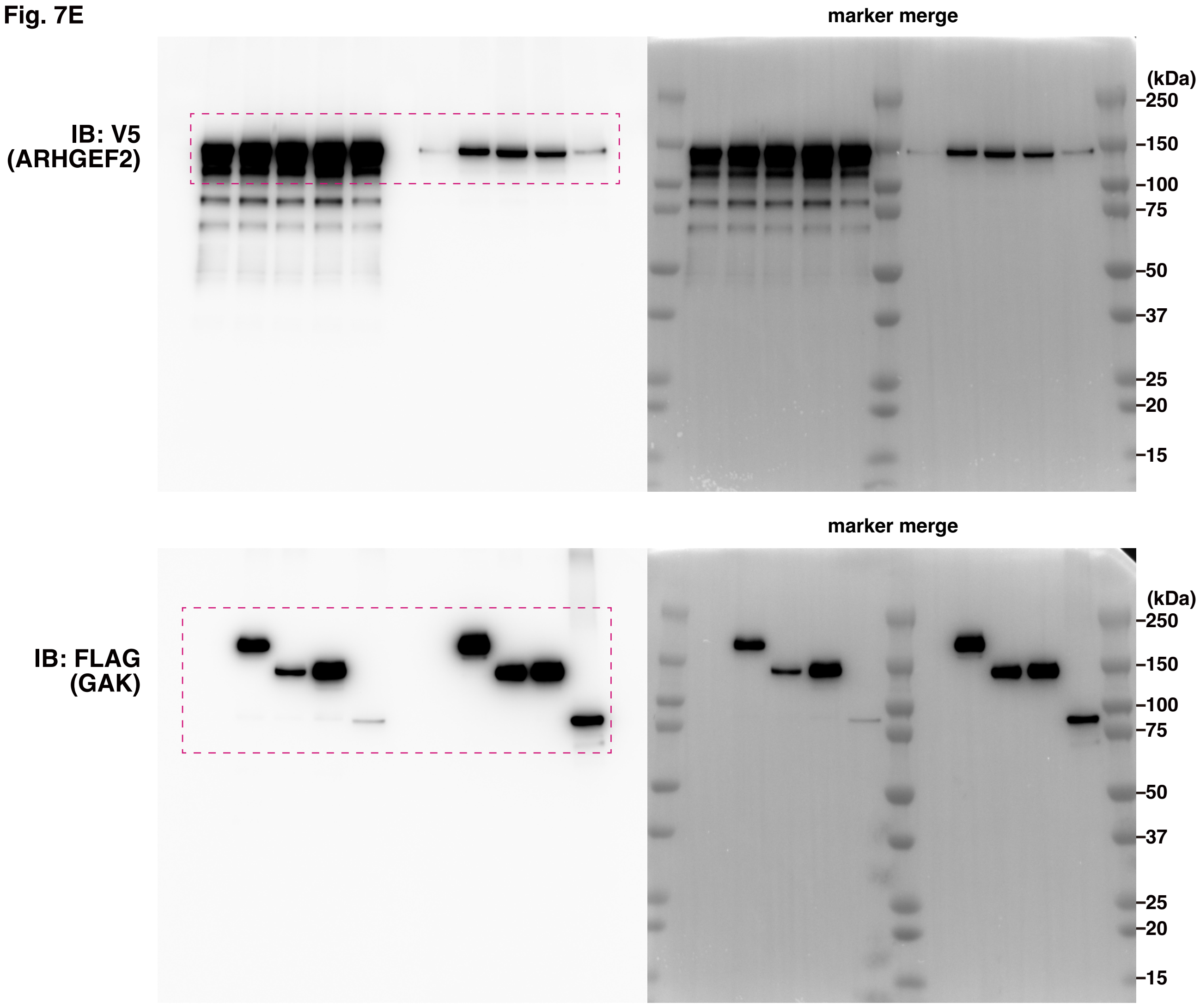

Fig. 7G

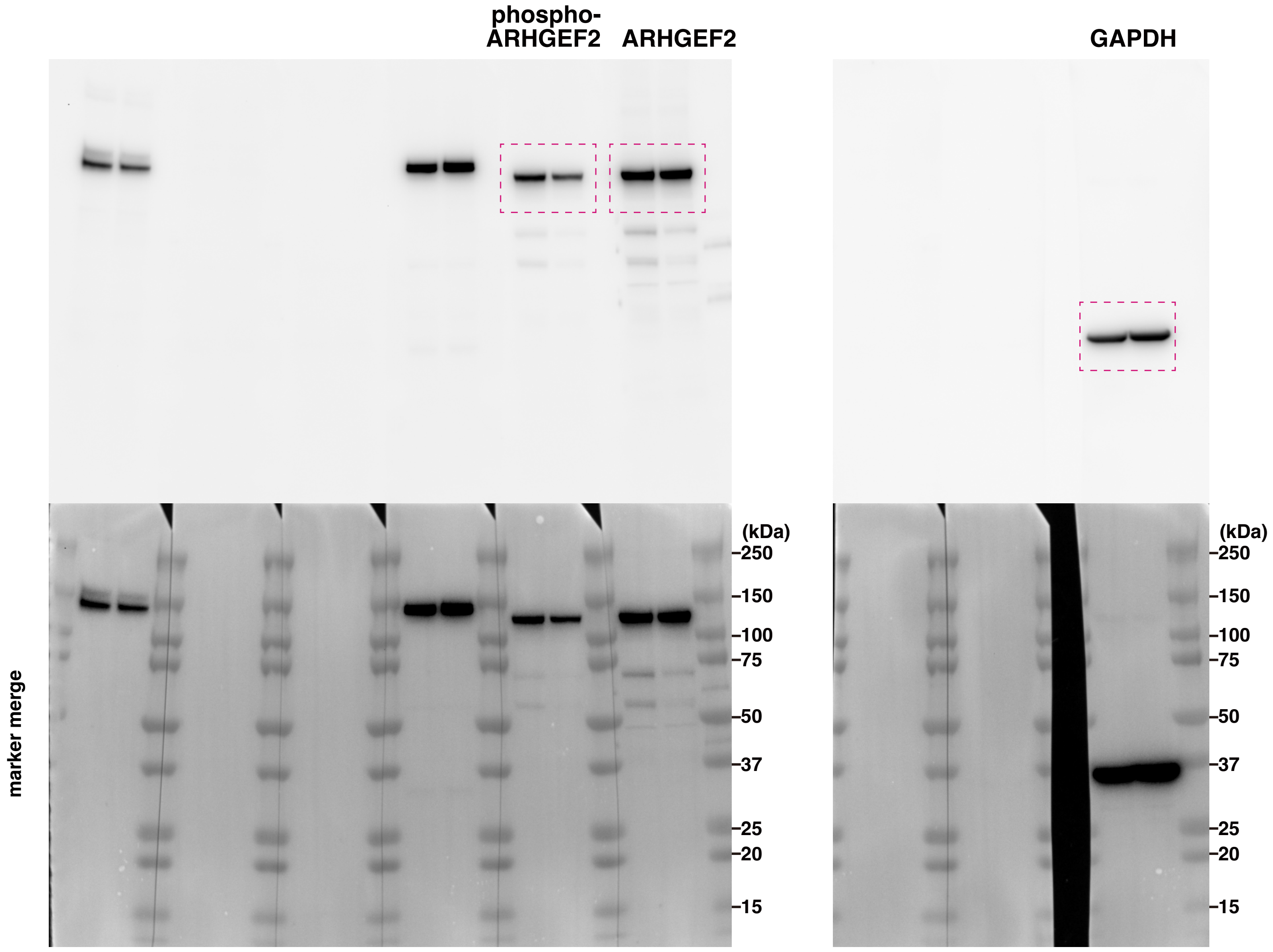

Journal of Cell Science • Supplementary information

(continued)

Fig. S1A

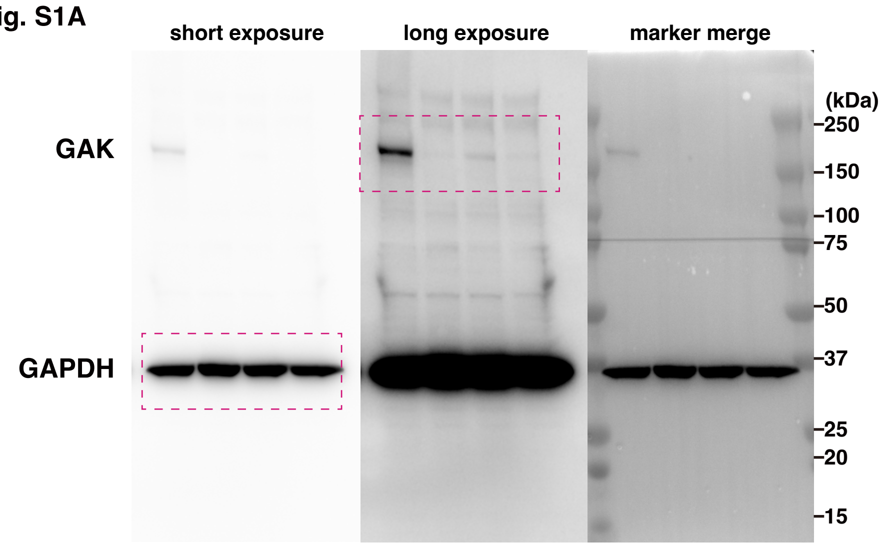

Fig. S1F

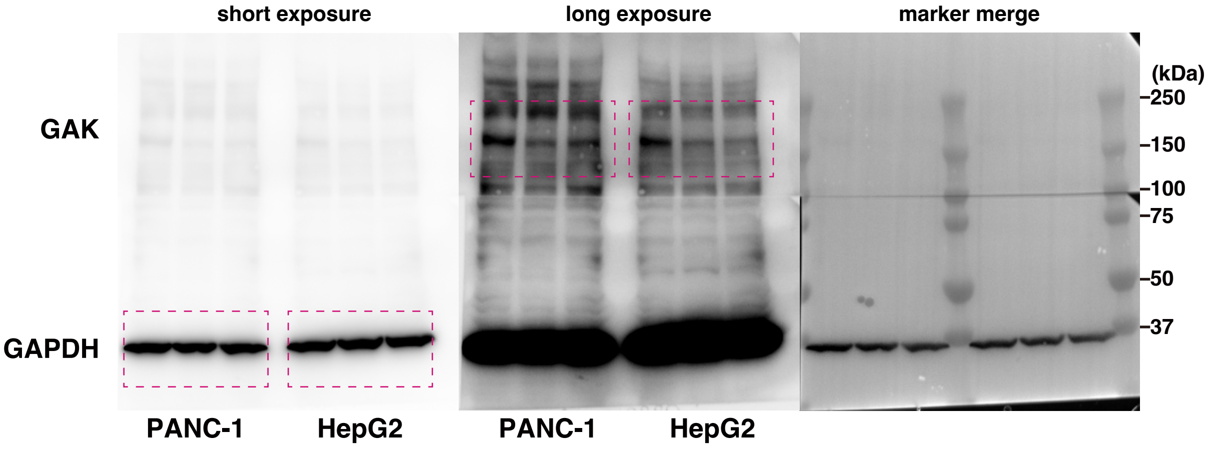

CAL27

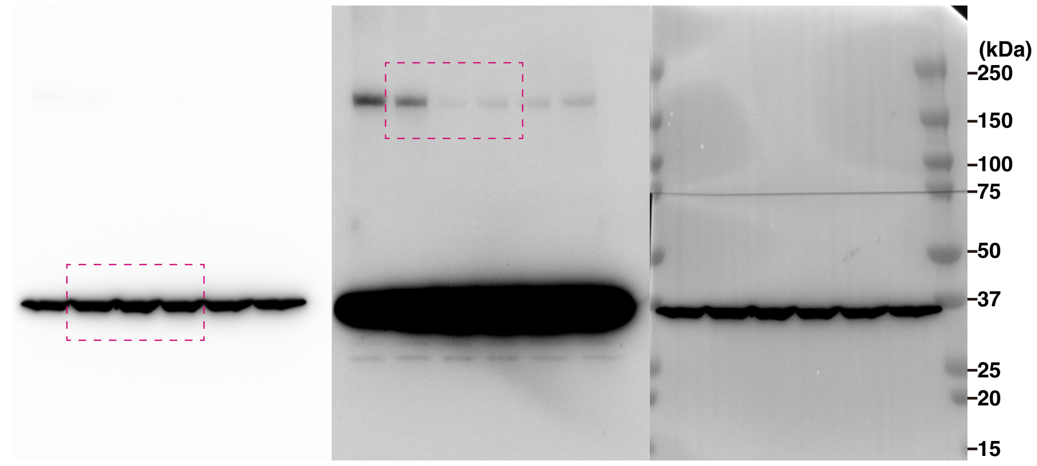

SH-SY5Y

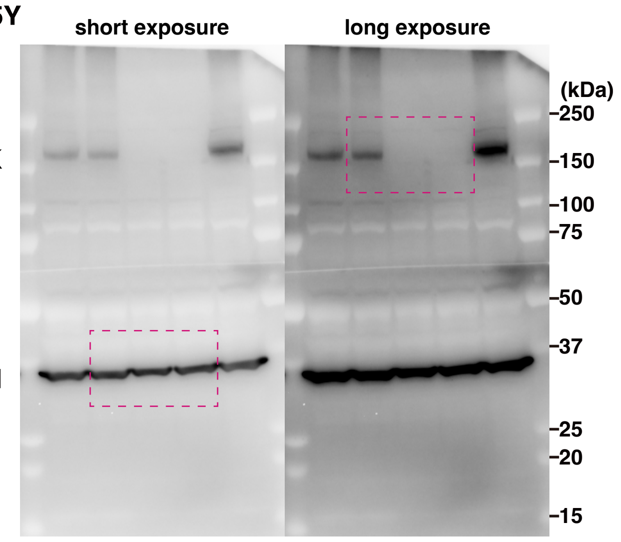

Journal of Cell Science • Supplementary information

(continued)

Fig. S2A

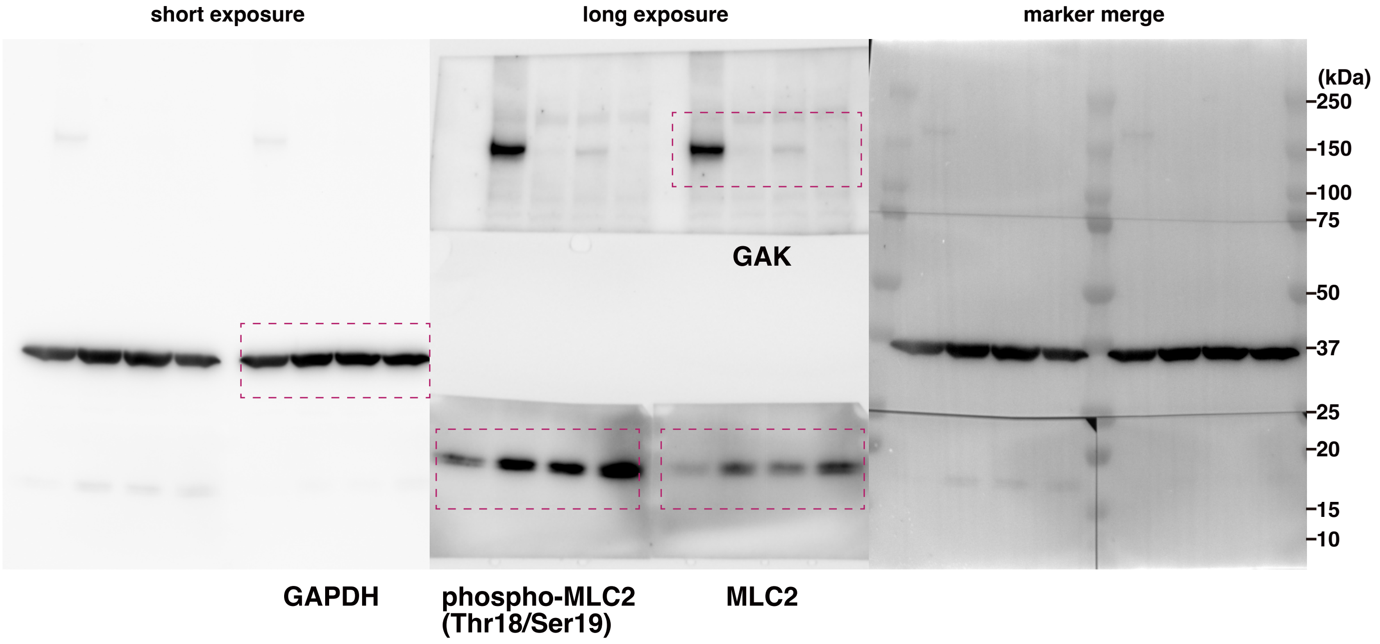

Fig. S2C

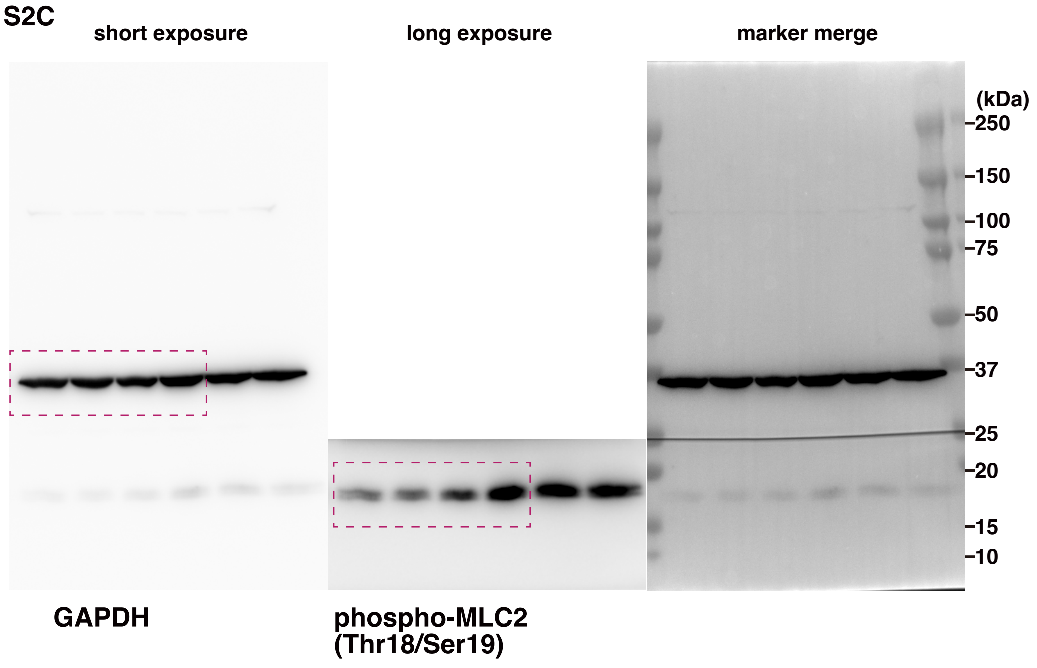

Fig. S2E

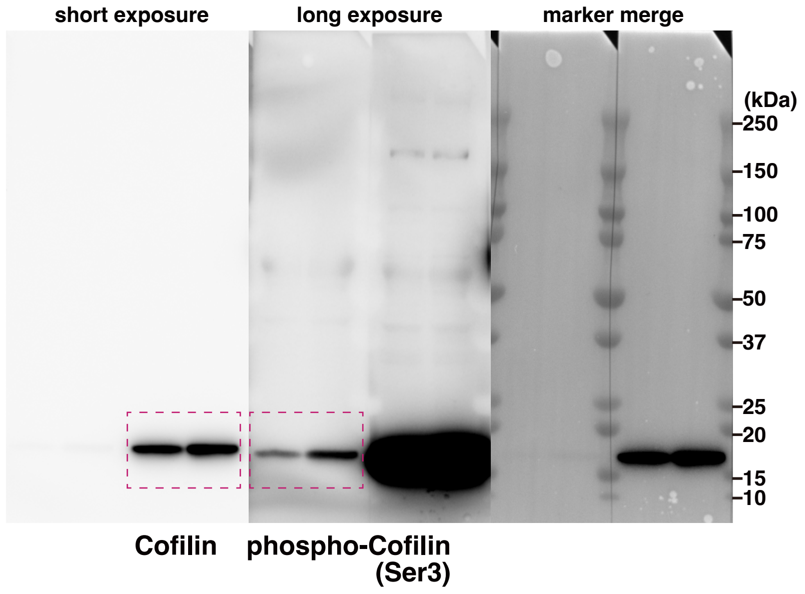

Journal of Cell Science • Supplementary information

(continued)

Fig. S3A

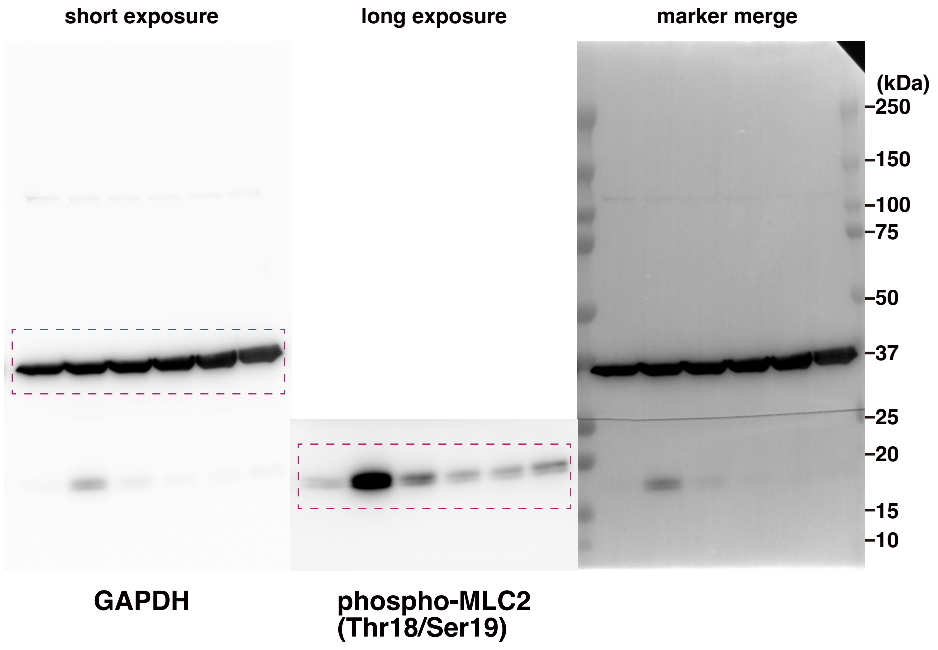

Fig. S3C

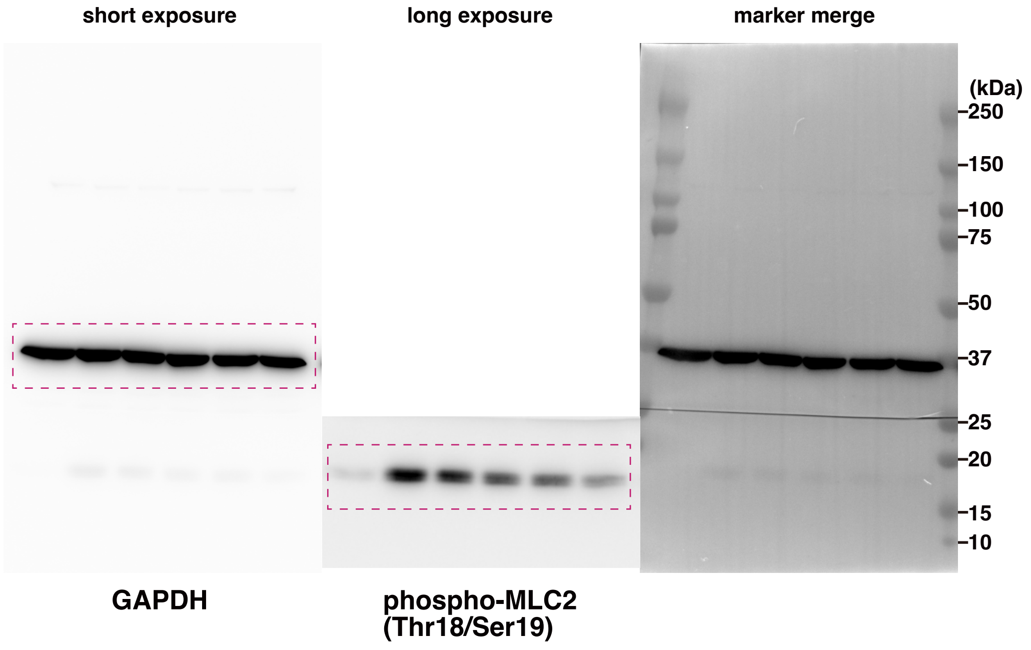

Journal of Cell Science • Supplementary information

Fig. S4A (continued)

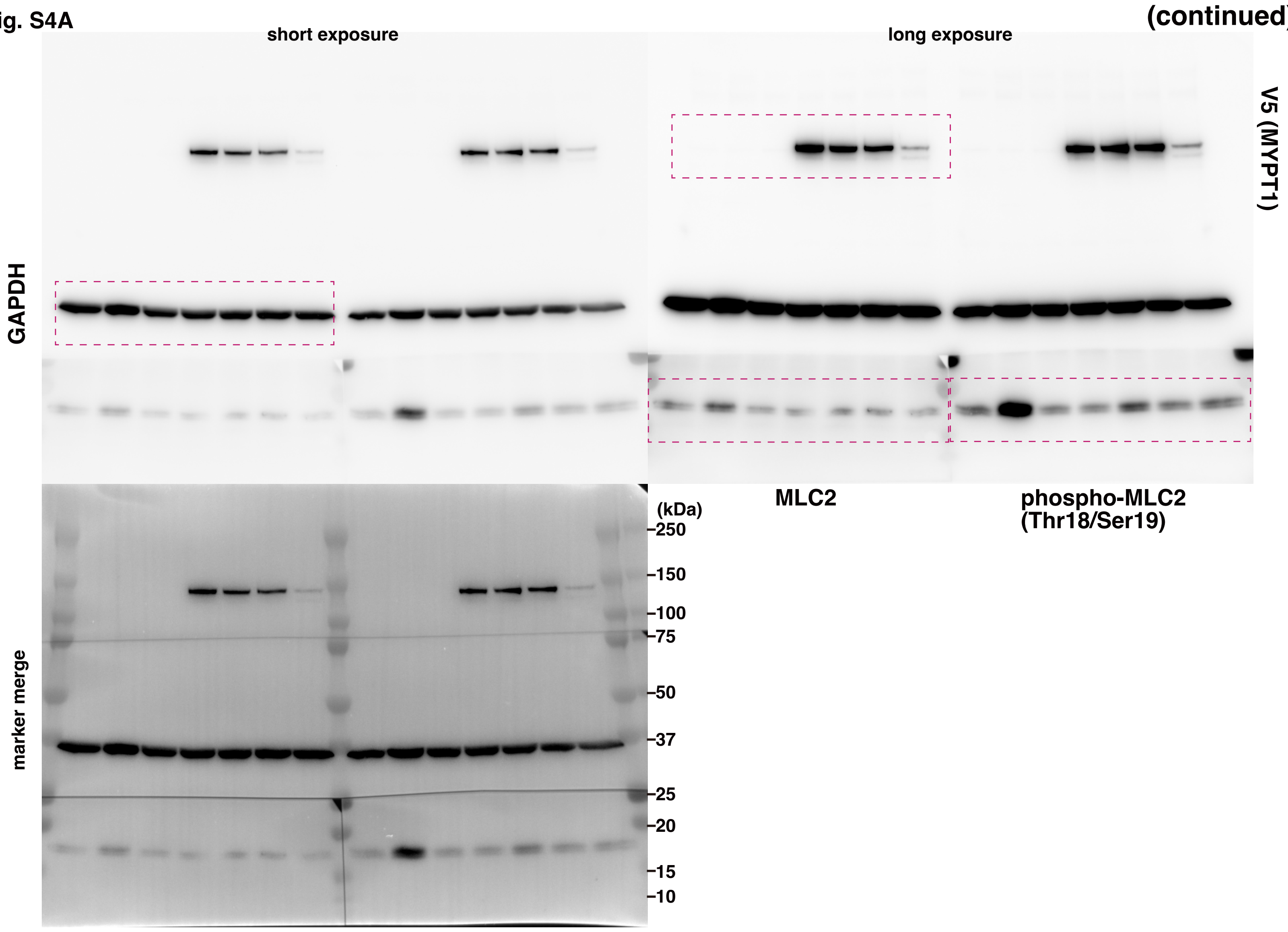

Fig. S5A

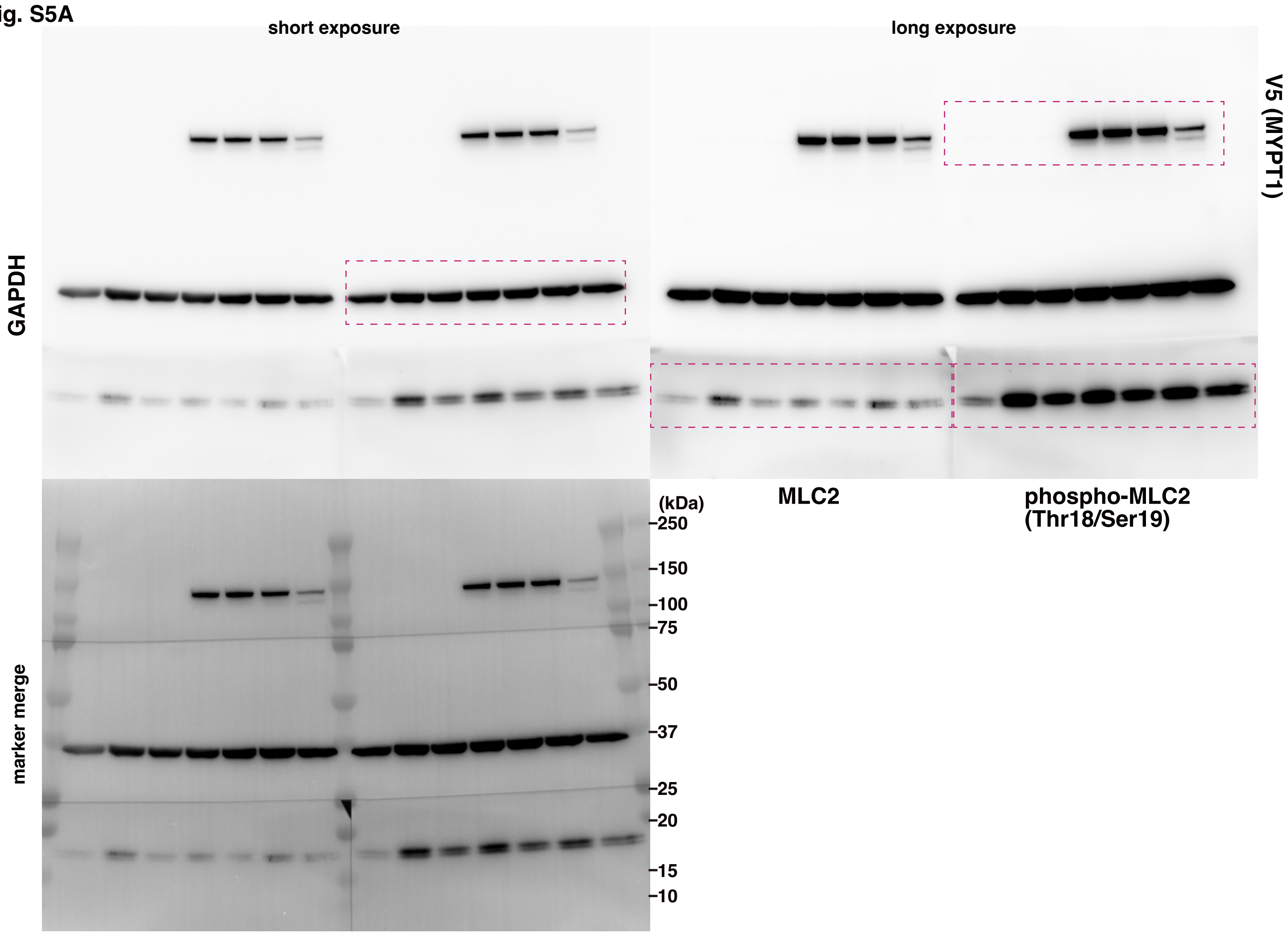

Journal of Cell Science • Supplementary information

(continued)

Fig. S6A

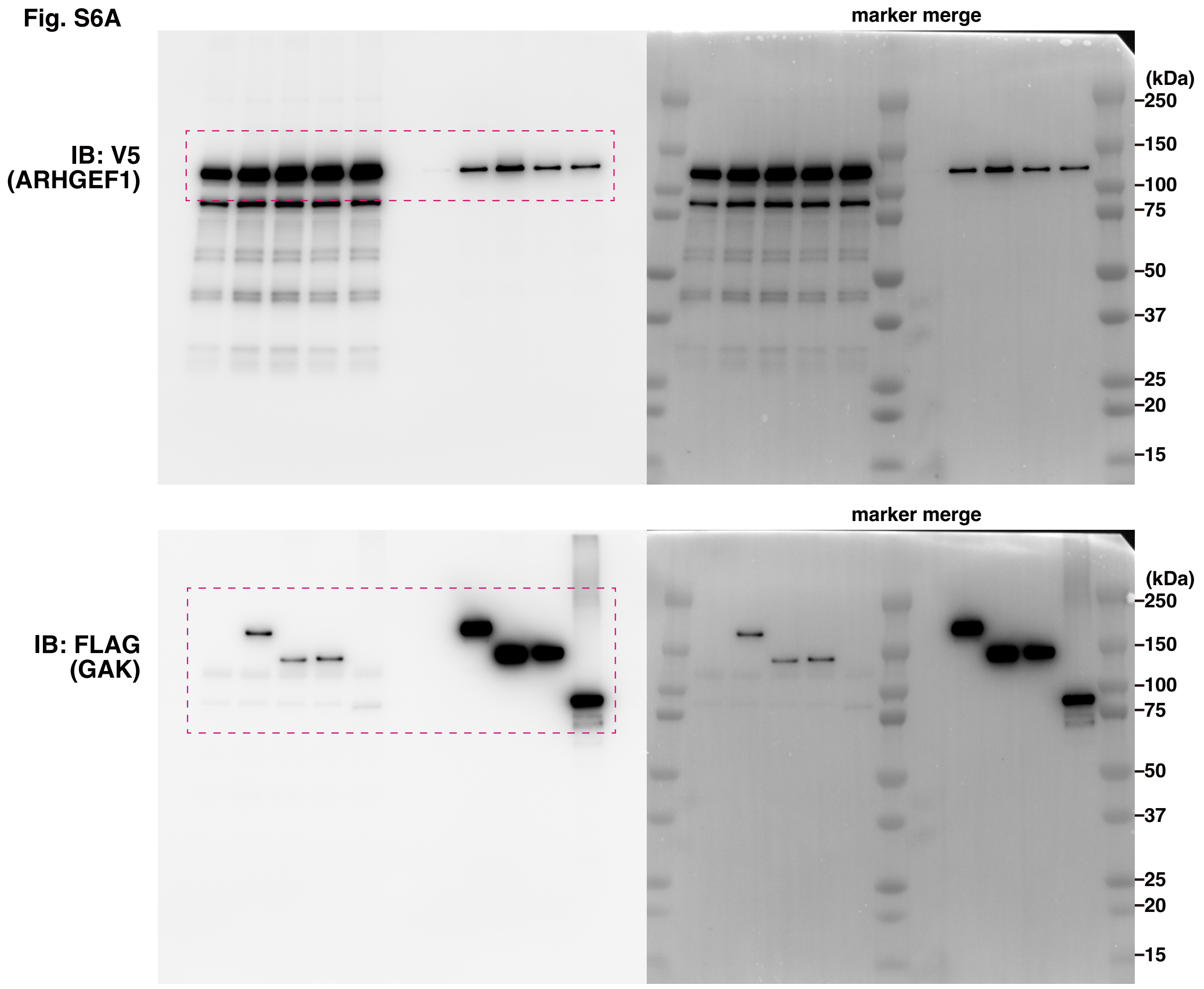

Fig. S6E

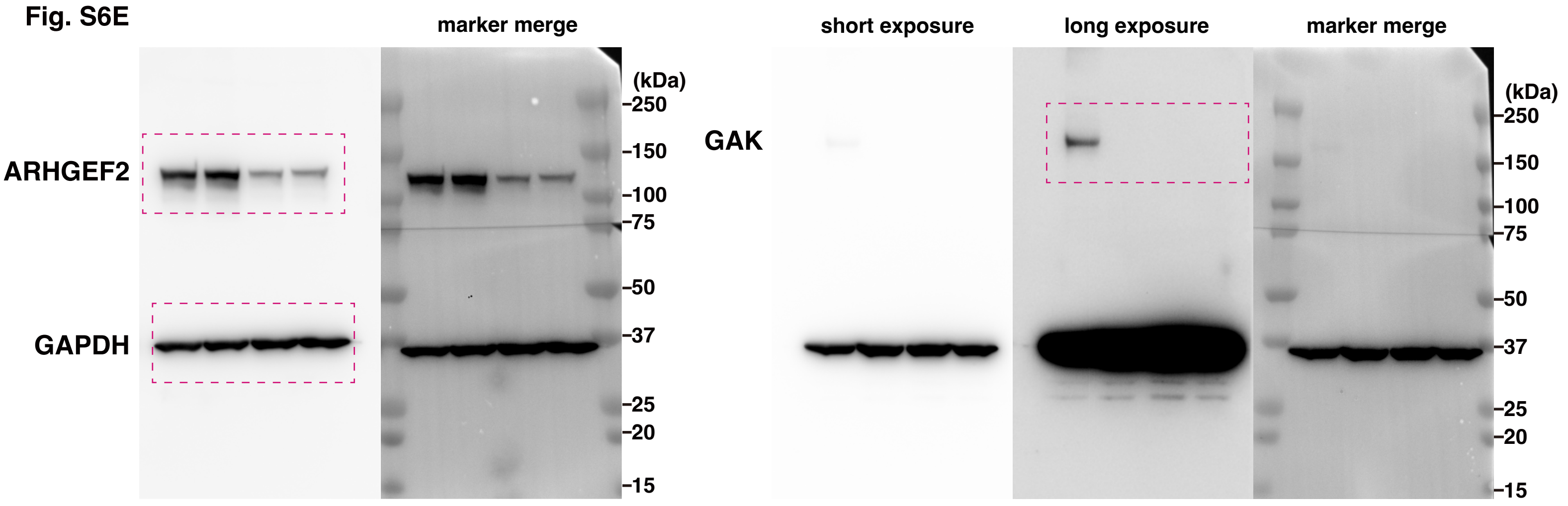

Fig. S6F

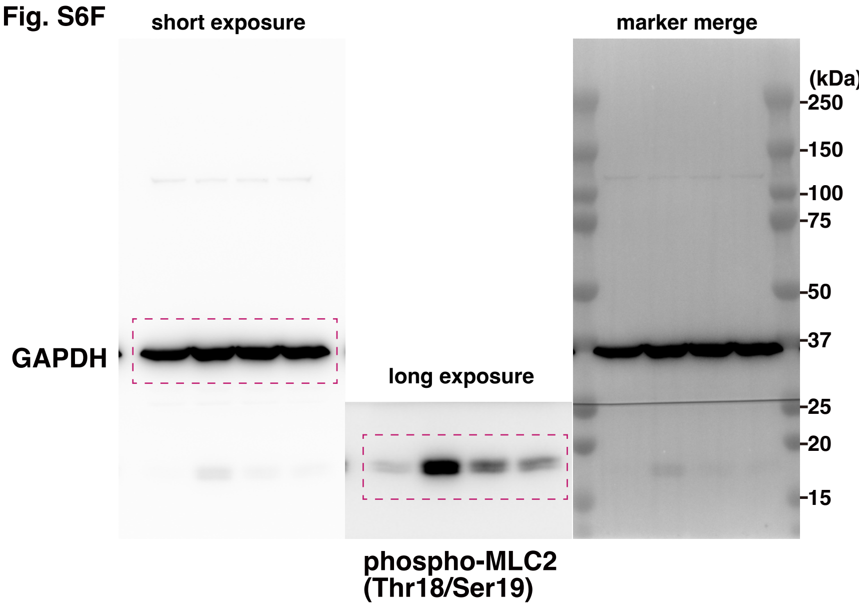

(continued)

Fig. S6H

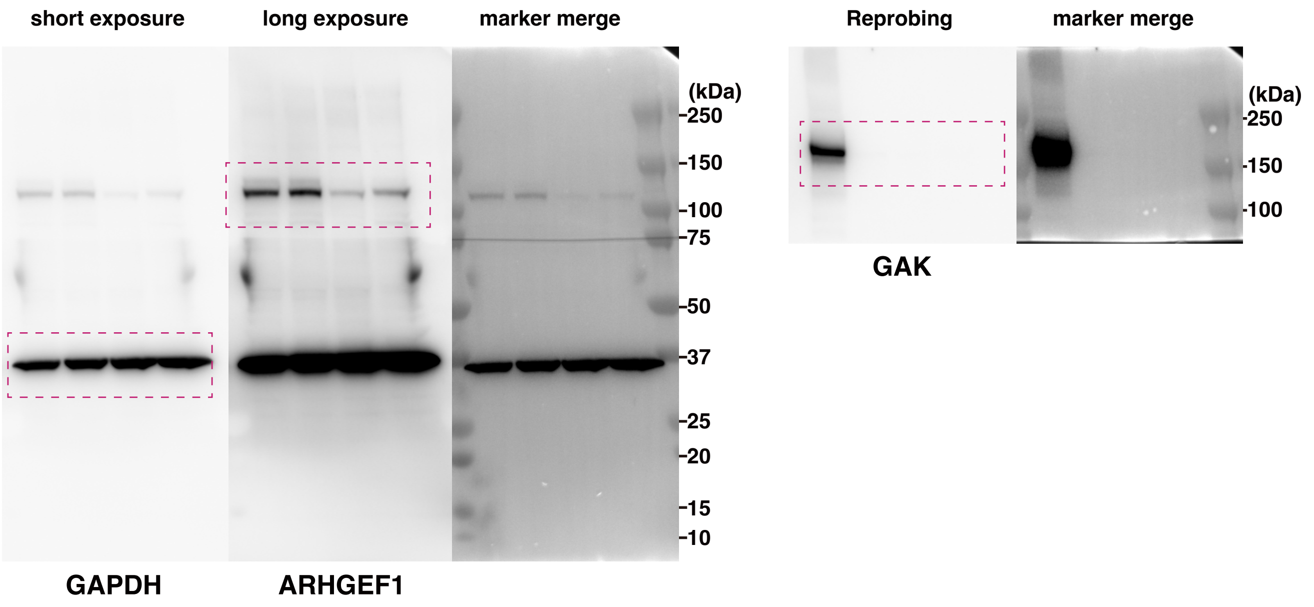

Fig. S6I

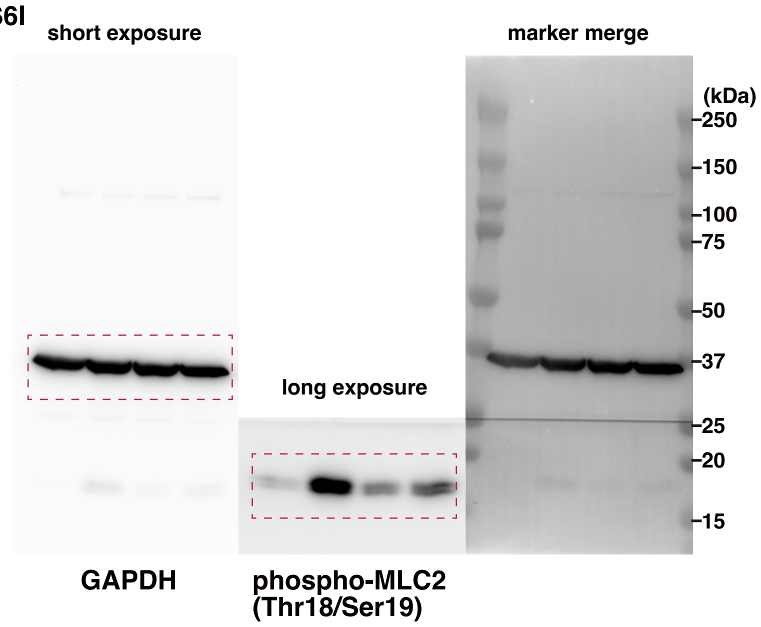

Fig. S7A

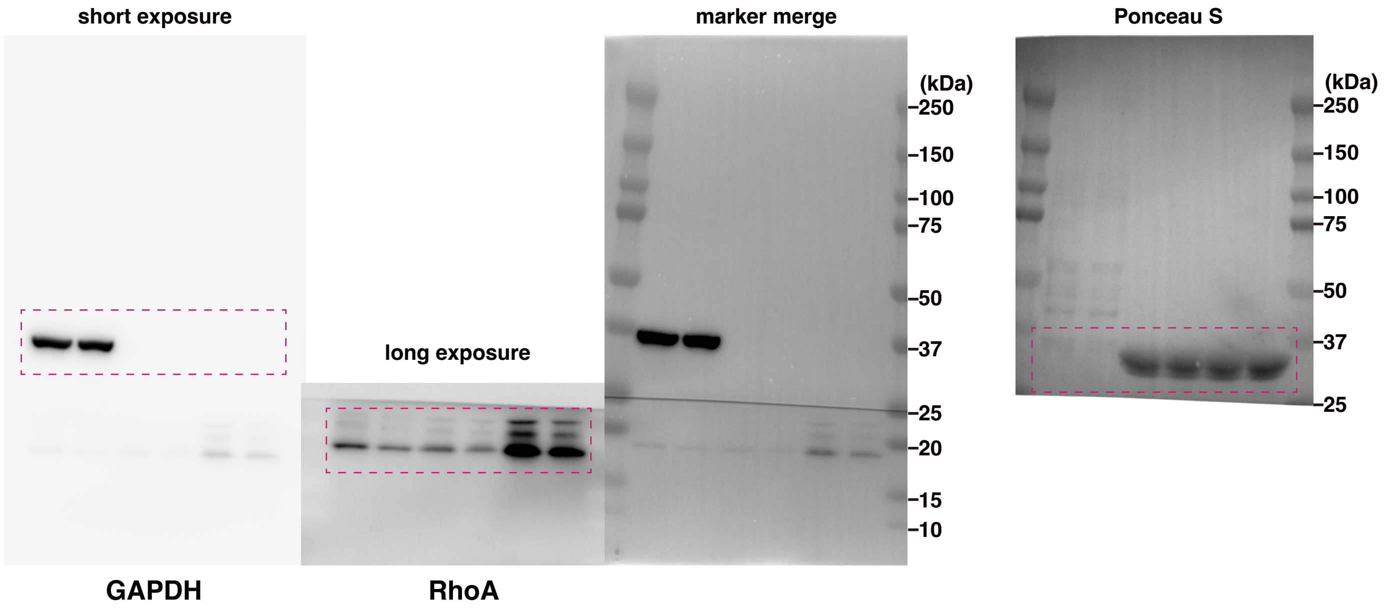

(continued)

Fig. S7F

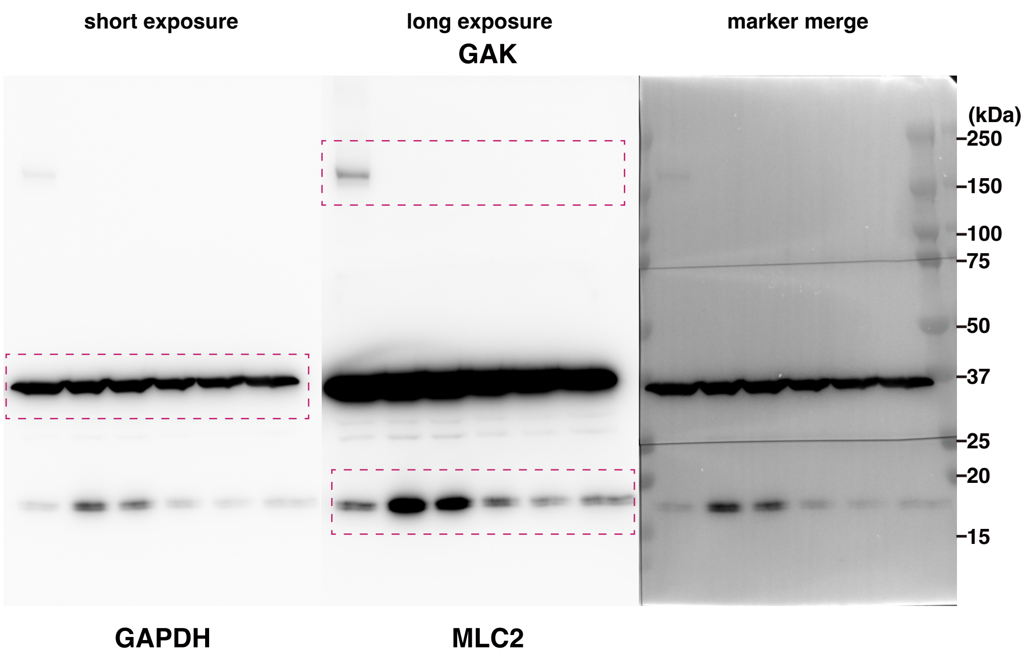

Fig. S9. Blot transparency. Original, uncropped immunoblot images from which the bands shown in the figures were derived.

Journal of Cell Science • Supplementary information

Table S1. Oligonucleotides used for site-directed mutagenesis of *GAK*.

Mutation		Sequence (5'→3')
K69A	Forward	GTGGCAGAGAGTATGCATTAGCGAGGCTATTATCCAATGAAG
	Reverse	CTTCATTGGATAATAGCCTCTTTAATGCATACTCTCTGCCAC
D173A	Forward	CGCCCATCATCCACAGGGCCCTCAAGGTTGAGAACTTG
	Reverse	CAAGTTCTCAACCTTGAGGTCCCTGTGGATGATGGGCG
delM1	Forward	GCGGCCGCCCGCAACGTGAAC
	Reverse	CAGCTCGCCCAGTTCCACCGTC
delM2	Forward	CCCAGGGACAGGCCGAGCCGG
	Reverse	CTGGATGACCTTGGAGGAGGTG
delM3	Forward	GGGGAGAGCCGCTGGACGCCC
	Reverse	GGGCTCCACCTCCACTTCCAG

Table S2. Oligonucleotides used for construction of lentiviral shRNA vectors.

Target gene		Sequence (5'→3')
GAK #1	Sense	CCGGAGCATTCCAAAGCCTCTGATTCTCGAGAATCAGAGGCTTTGGAATGCTTTTTTG
GAK #1	Antisense	AATTCAAAAAAAGCATTCCAAAGCCTCTGATTCTCGAGAATCAGAGGCTTTGGAATGCT
GAK #2	Sense	CCGGCCTCTGATTGTTGTTTCCTTTCTCGAGAAAGGAAACAACAATCAGAGGTTTTTG
GAK #2	Antisense	AATTCAAAAACCTCTGATTGTTGTTTCCTTTCTCGAGAAAGGAAACAACAATCAGAGG
ROCK1 #1	Sense	CCGGCGGGTTGTTCAGATTGAGAAACTCGAGTTTCTCAATCTGAACAACCCGTTTTTG
ROCK1 #1	Antisense	AATTCAAAAACGGGTTGTTCAGATTGAGAAACTCGAGTTTCTCAATCTGAACAACCCG
ROCK1 #2	Sense	CCGGGAGGTAAATGAACACAAAGTACTCGAGTACTTTGTGTTCATTTACCTCTTTTTG
ROCK1 #2	Antisense	AATTCAAAAAGAGGTAAATGAACACAAAGTACTCGAGTACTTTGTGTTCATTTACCTC
ROCK2	Sense	CCGGGCACAGTTTGAGAAGCAGCTACTCGAGTAGCTGCTTCTCAAACTGTGCTTTTTG
ROCK2	Antisense	AATTCAAAAAGCACAGTTTGAGAAGCAGCTACTCGAGTAGCTGCTTCTCAAACTGTGC
ARHGEF2 #1	Sense	CCGGGCGGCGAATTAAGATGGAGTTCTCGAGAACTCCATCTTAATTCGCCGCTTTTTG
ARHGEF2 #1	Antisense	AATTCAAAAAGCGGCGAATTAAGATGGAGTTCTCGAGAACTCCATCTTAATTCGCCGC
ARHGEF2 #2	Sense	CCGGCGTAGGCAATTAGAGATCGAACTCGAGTTCGATCTCTAATTGCCTACGTTTTTG
ARHGEF2 #2	Antisense	AATTCAAAAACGTAGGCAATTAGAGATCGAACTCGAGTTCGATCTCTAATTGCCTACG
ARHGEF1 #1	Sense	CCGGCACCGATCACAAAGCCTTCTACTCGAGTAGAAGGCTTTGTGATCGGTGTTTTTG
ARHGEF1 #1	Antisense	AATTCAAAAACACCGATCACAAAGCCTTCTACTCGAGTAGAAGGCTTTGTGATCGGTG
ARHGEF1 #2	Sense	CCGGCCATCTCTACCGACGAAGAAACTCGAGTTTCTTCGTCGGTAGAGATGGTTTTTG
ARHGEF1 #2	Antisense	AATTCAAAAACCATCTCTACCGACGAAGAAACTCGAGTTTCTTCGTCGGTAGAGATGG
MYL9 #1	Sense	CCGGCGCCAAGGATAAAGACGACTACTCGAGTAGTCGTCTTTATCCTTGGCGTTTTTG
MYL9 #1	Antisense	AATTCAAAAACGCCAAGGATAAAGACGACTACTCGAGTAGTCGTCTTTATCCTTGGCG
MYL9 #2	Sense	CCGGCATTGATAAGAAAGGCAACTTCTCGAGAAGTTGCCTTTCTTATCAATGTTTTTG
MYL9 #2	Antisense	AATTCAAAAACATTGATAAGAAAGGCAACTTCTCGAGAAGTTGCCTTTCTTATCAATG
MYL9 #3	Sense	CCGGGATGTGATTCGCAACGCCTTTCTCGAGAAAGGCGTTGCGAATCACATCTTTTTG
MYL9 #3	Antisense	AATTCAAAAAGATGTGATTCGCAACGCCTTTCTCGAGAAAGGCGTTGCGAATCACATC
Non-targeting	Sense	CCGGCAACAAGATGAAGAGCACCAACTCGAGTTGGTGCTCTTCATCTTGTTGTTTTTG
Non-targeting	Antisense	AATTCAAAAACAACAAGATGAAGAGCACCAACTCGAGTTGGTGCTCTTCATCTTGTTG

Table S3. Oligonucleotides used for construction of mammalian expression vectors.

Target gene		Sequence (5'→3')
MYPT1	Forward	CACCGCGGCCGCCACCATGAAGATGGCGGACGCGAAGC
MYPT1	Reverse	AGCTGGCGCGCCCTTTGGAAAGTTTGCTTATAACTCTGATC
MPRIP	Forward	CACCGCGGCCGCCACCATGTCGGCAGCCAAGGAGAACC
MPRIP	Reverse	AGCTGGCGCGCCCGTCTTTCTTCAAGTCCCTGGATTC
PPP1CA	Forward	CACCGCGGCCGCCACCATGTCCGACAGCGAGAAGCTCAAC
PPP1CA	Reverse	AGCTGGCGCGCCCTTTCTTGGCTTTGGCGGAATTGCG
RHOA	Forward	CACCGCGGCCGCCACCATGGCTGCCATCCGGAAGAAAC
RHOA	Reverse	AGCTGGCGCGCCCCAAGACAAGGCACCCAGATTTTTTC
ROCK1	Forward	CACCGCGGCCGCCACCATGTCGACTGGGGACAGTTTTG
ROCK1	Reverse	AGCTGGCGCGCCCACTAGTTTTTCCAGATGTATTTTTGAC
ROCK2	Forward	CACCGCGGCCGCCACCATGAGCCGGCCCCCGCCGAC
ROCK2	Reverse	AGCTGGCGCGCCCGCTAGGTTTGTTTGGGGCAAGC
ARHGEF1	Forward	CACCGCGGCCGCCACCATGGCTTCTCTTTCCACCTGGAG
ARHGEF1	Reverse	AGCTGGCGCGCCCAGTGCAGCCAGGCTGGGGGAC
ARHGEF2	Forward	CACCGCGGCCGCCACCATGTCTCGGATCGAATCCCTCAC
ARHGEF2	Reverse	AGCTGGCGCGCCCGCTCTCGGAGGCTACAGCCTC
ARHGEF7	Forward	CACCGCGGCCGCCACCATGACCGATAATAGCAACAATCAAC
ARHGEF7	Reverse	AGCTGGCGCGCCCTAGATTGGTCTCATCCCAGGCAG
ARHGEF9	Forward	CACCGCGGCCGCCACCATGACGTTGCTGATCACTGGAG
ARHGEF9	Reverse	AGCTGGCGCGCCCTTTTTTGAAGGGGGTTAACCTGC
ARHGEF11	Forward	CACCGCGGCCGCCACCATGAGTGTAAGGTTACCCCAGAG
ARHGEF11	Reverse	AGCTGGCGCGCCCTGGTCCTGGTGACGCGGCTGC
ARHGEF28	Forward	CACCGCGGCCGCCACCATGGAGTTGAGCTGCAGCGAAG
ARHGEF28	Reverse	AGCTGGCGCGCCCGAGGTAAACAATATTTTCTTTGGCTC

Journal of Cell Science • Supplementary information

Table S4. Oligonucleotides used for construction of *E.coli* expression vectors.

Target region		Sequence (5’→3’)
GAK (25-335)	Forward	CACCGGTACCGACTACAAGGATGACGACGACAAGCAGAGTGACTTCGTGGGGCAG
	Reverse	AGCTGAATTCATTCTGCTCCAGGAGCTCTGTG
EGFP	Forward	CACCGAATTCATGGTGAGCAAGGGCGAGGAG
	Reverse	AGCTGTCGACTTACTTGTACAGCTCGTCCATGC
AP2M1 (1-164)	Forward	AGCTGCGGCCGCCTCTCGCCGCCAGCCAATCTG
	Reverse	CACCGTCGACTCATGATTGGAGGCTTATTCATCTATAATC
ARHGEF2 (439-582)	Forward	CACCGTCGACTCGGGGCCCGTCTGCAGGAGATC
	Reverse	AGCTGCGGCCGCAATCAGGGGGAAGTCCTCCCTGG
ARHGEF2 (862-986)	Forward	CACCGTCGACTCGCCCTGGGCCAGACCGAGCCAC
	Reverse	AGCTGCGGCCGCTTAGCTCTCGGAGGCTACAGCC
MPRIP (673-976)	Forward	CACCGTCGACTCCACGAGCTGACCTCTCTGCTCG
	Reverse	AGCTGCGGCCGCCTTCTCCCCCAGTGCTTCCGTTG
MYPT1 (300-471)	Forward	CACCGTCGACTCCCACTAATTGAATCAACAGCAAATATGG
	Reverse	AGCTGCGGCCGCAGCTGAACGTGTAACACCTGCAG
MYPT1 (697-902)	Forward	CACCGTCGACTCCAGGGAGTGACATTAACTGATCTTC
	Reverse	AGCTGCGGCCGCATCATATCGATCACCAGCTGATGTAG
Rhotekin (7-89)	Forward	CACCGGATCCCCGGCCAGGGGCTCCGCCCTGGAG
	Reverse	AGCTGAATTCGCTTGTCTTCCCCAGCACCTGC

Table S5. Oligonucleotides used for site-directed mutagenesis of *MYPT1*.

Mutation		Sequence (5'→3')
T443A	Forward	GGAGGTTAGGACTTAGAAAGGCGGGCAGCTAT GGTGCACTTG
	Reverse	CAAGTGCACCATAGCTGCCCGCCTTTCTAAGTC CTAACCTCC
T443D	Forward	GGAGGTTAGGACTTAGAAAGGATGGCAGCTAT GGTGCACTTG
	Reverse	CAAGTGCACCATAGCTGCCATCCTTTCTAAGTC CTAACCTCC
T443E	Forward	GGAGGTTAGGACTTAGAAAGGAGGGCAGCTAT GGTGCACTTG
	Reverse	CAAGTGCACCATAGCTGCCCTCCTTTCTAAGTC CTAACCTCC

Table S6. Oligonucleotides used for qPCR.

Target gene		Sequence (5'→3')
MYL9	Forward	GAGCCCAAGCGCCTTCTC
MYL9	Reverse	GGATCTGGGACTGGTCAAAC
MYL12A	Forward	CACCATGTCGAGCAAAAGAA
MYL12A	Reverse	GTTGGATTCTTCCCCAATGA
MYL12B	Forward	CAGAGATGGCTTCATCGACA
MYL12B	Reverse	GCATCAGTGGGATTCTTCCCTAGA
MYL5	Forward	TGAACCTGTTTGGGGAGAAG
MYL5	Reverse	TCTTGTCAGCCTGGGACATC
MYL6	Forward	AGTGTGGGGATGTGATGAGG
MYL6	Reverse	TGGGCAGAAAGTGCTCAAAG
MYL6B	Forward	TCACCACCCTTGGAGAGAAG
MYL6B	Reverse	AGCACTCAGACGCTTAGGATG
MYH9	Forward	TTCAAGAAGGAGCGGAACAC
MYH9	Reverse	ATACCCAAGAGATGGGACACC
MYH10	Forward	AGGTCAGCACCCTGAAGAAC
MYH10	Reverse	TCTGTGTCATCGTCGGAGAG

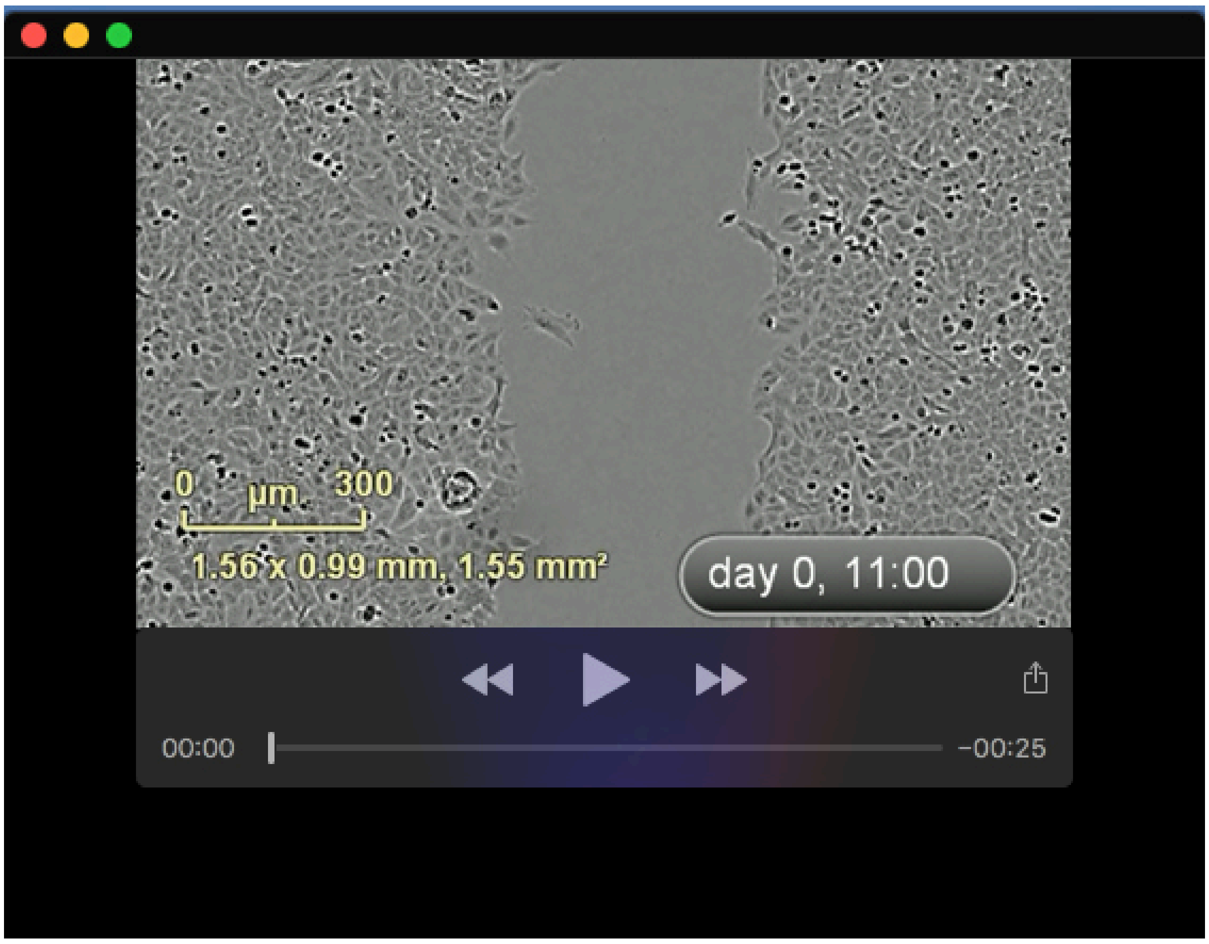

Movie 1. Time-lapse microscopy of A549 WT cells in a wound-healing assay.
A video generated from phase-contrast images of A549 WT cells acquired hourly for 48 h after scratching using the IncuCyte ZOOM system.

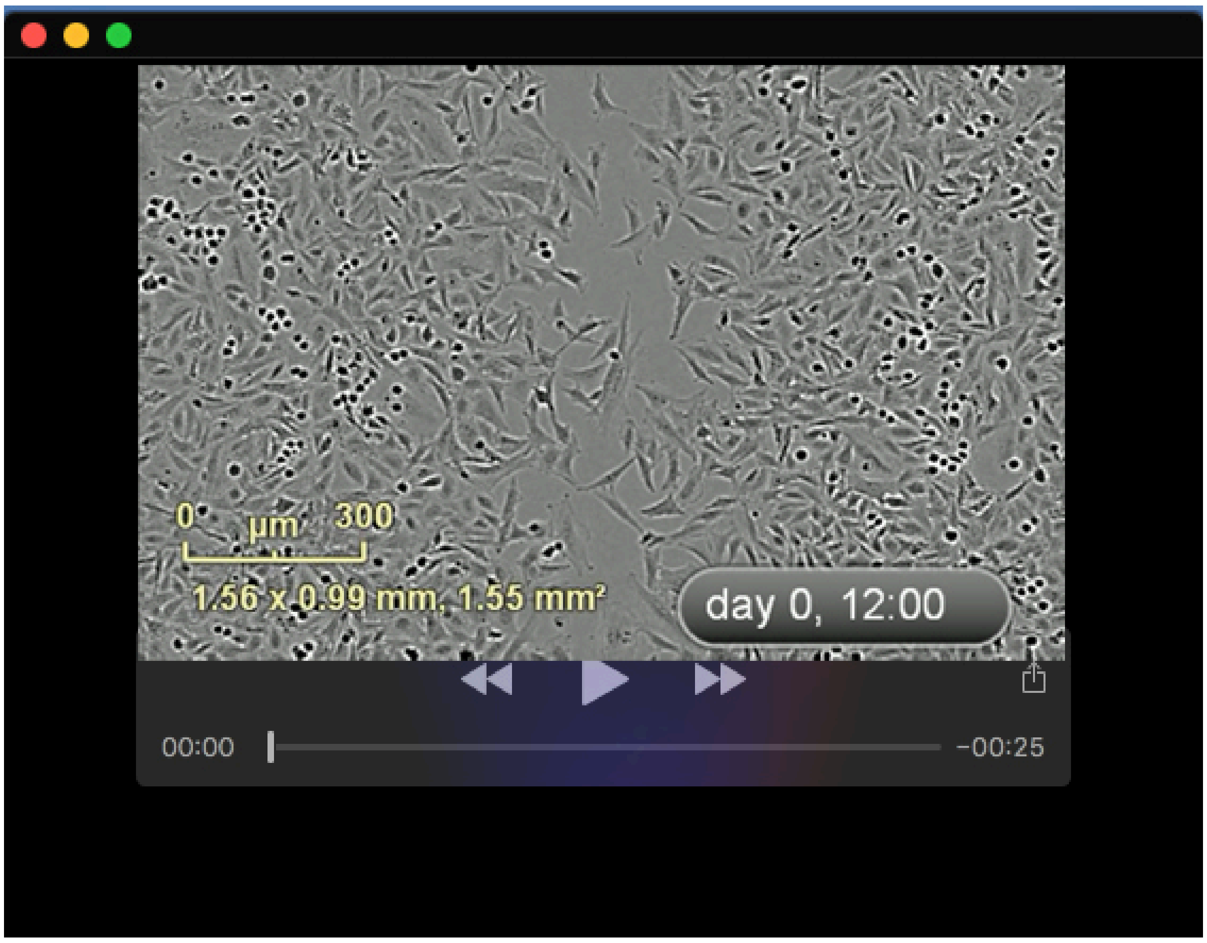

Movie 2. Time-lapse microscopy of A549 GAK-KO cells in a wound-healing assay.
A video generated from phase-contrast images of A549 GAK-KO cells acquired hourly for 48 h after scratching, using the IncuCyte ZOOM system.

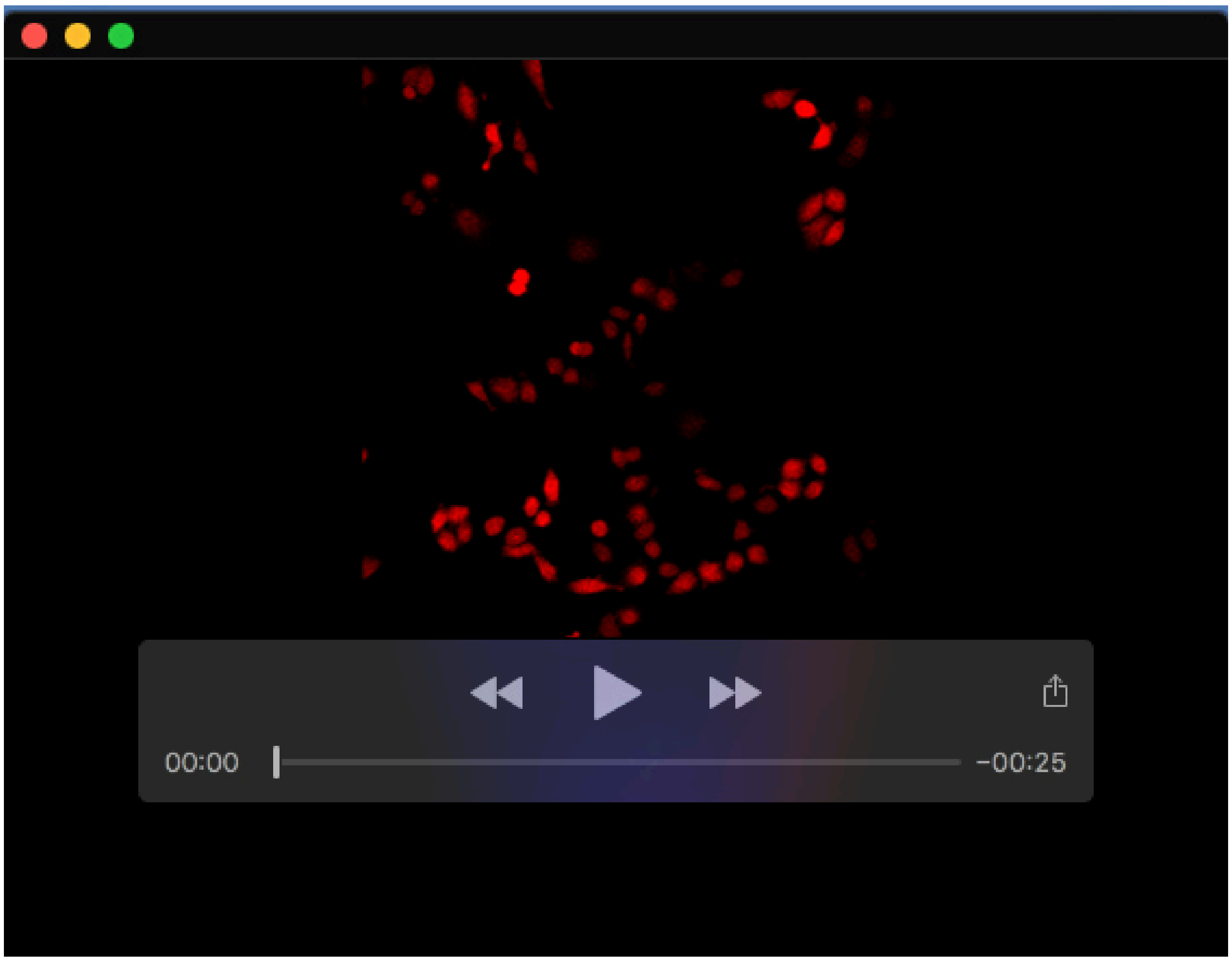

Movie 3. Time-lapse microscopy of A549 WT cells in a random migration assay.
A video created from fluorescent images of A549/tdTomato cells acquired every 15 min for 24 h using the Zeiss LSM700 microscope.

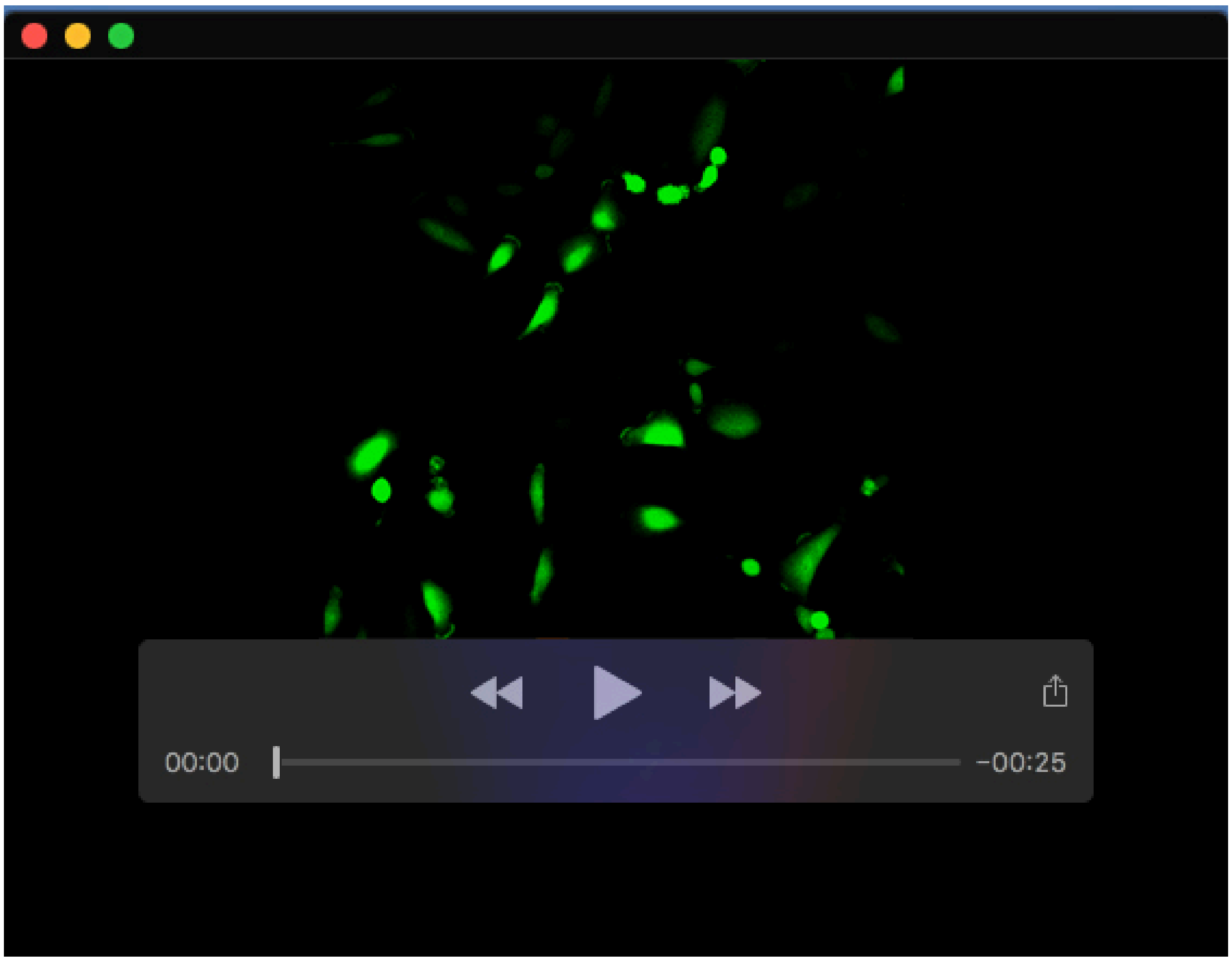

Movie 4. Time-lapse microscopy of A549 GAK-KO cells in a random migration assay.
A video created from fluorescent images of A549 GAK-KO/EGFP cells acquired every 15 min for 24 h using the Zeiss LSM700 microscope.

Journal of Cell Science • Supplementary information
